# Myc-induced nuclear antigen constrains a latent intestinal epithelial cell-intrinsic anthelmintic pathway

**DOI:** 10.1371/journal.pone.0211244

**Published:** 2019-02-26

**Authors:** Meenu R. Pillai, Belgacem Mihi, Kenji Ishiwata, Kiminori Nakamura, Naoya Sakuragi, David B. Finkelstein, Maureen A. McGargill, Toshinori Nakayama, Tokiyoshi Ayabe, Mathew L. Coleman, Mark Bix

**Affiliations:** 1 St. Jude Children’s Research Hospital, Memphis, Tennessee, United States of America; 2 Department of Innovative Medicine, Graduate School of Medicine and Institute for Global Prominent Research, Chiba University, Chiba, Japan; 3 Department of Tropical Medicine, The Jikei University School of Medicine, Tokyo, Japan; 4 Department of Cell Biological Science, Graduate School of Life Science, Hokkaido University, Sapporo, Hokkaido, Japan; 5 Department of Immunology, Graduate School of Medicine, Chiba University, Chiba, Japan; 6 Institute of Cancer and Genomic Sciences, University of Birmingham, Birmingham, United Kingdom; University of Manchester, UNITED KINGDOM

## Abstract

Expulsion of parasitic gastrointestinal nematodes requires diverse effector mechanisms coordinated by a Th2-type response. The evolutionarily conserved JmjC protein; Myc Induced Nuclear Antigen (Mina) has been shown to repress IL4, a key Th2 cytokine, suggesting Mina may negatively regulate nematode expulsion. Here we report that expulsion of the parasitic nematode *Trichuris muris* was indeed accelerated in Mina deficient mice. Unexpectedly, this was associated not with an elevated Th2- but rather an impaired Th1-type response. Further reciprocal bone marrow chimera and conditional KO experiments demonstrated that retarded parasite expulsion and a normal Th1-type response both required Mina in intestinal epithelial cells (IECs). Transcriptional profiling experiments in IECs revealed anti-microbial α-defensin peptides to be the major target of Mina-dependent retention of worms in infected mice. In vitro exposure to recombinant α-defensin peptides caused cytotoxic damage to whipworms. These results identify a latent IEC-intrinsic anthelmintic pathway actively constrained by Mina and point to α-defensins as important effectors that together with Mina may be attractive therapeutic targets for the control of nematode infection.

## Introduction

Over 2 billion people are chronically infected with parasitic gastrointestinal (GI) nematodes, extracting a massive global toll in morbidity and mortality [1]; in addition, the rapid spread of drug resistance in livestock GI nematodes has created a global economic crisis that portends a further humanitarian one [2–10]. Expulsion of GI nematodes requires the host T helper 2 (Th2)-type response to mobilize multiple GI effector mechanisms including goblet cell hyperplasia and increased epithelial cell turnover [11]. However, the full panoply of effector mechanisms remains still to be elucidated. We have shown that the JmjC protein Mina can repress transcription of IL4, a key Th2 cytokine, suggesting a possible negative regulatory role in GI nematode expulsion [12]. To gain further insight, we explored genetic models of Mina deficiency during infection with the parasitic whipworm *Trichuris muris* (TM) that models human infection with the soil transmitted helminth *Trichuris trichiura* [13]. Unexpectedly, we discovered Mina acts in intestinal epithelial cells (IECs) rather than CD4 T cells to constrain parasite expulsion by a novel mechanism associated with mobilization of anti-microbial α-defensins that can directly inflict damage upon parasites and with repression of the Th1 response that antagonizes worm expulsion. Thus, therapeutic inhibition of Mina in intestinal epithelial cells could unleash a latent host-protective anthelmintic response.

## Results

### Mina deficient mice develop normally

In order to explore the physiological role of Mina in host resistance to nematode parasites, we generated germline null (knockout, ‘KO’) and floxed *Mina* alleles targeting exons 3 and 4 (encoding the catalytic JmjC domain; S1A and S1B Fig). Mina protein (S1C Fig) was undetectable in Mina KO mice, demonstrating that exon 3–4 deletion creates a null allele. Despite this, germline Mina KO mice were viable, fertile (in both genders) and appeared grossly normal from birth onward, consistent with a previous report of Mina deficient mice in which exon 2 was replaced with a neomycin cassette [14]. Importantly, we also confirmed that the expression of Mina is ablated by qRT-PCR in uninfected and infected cecum tissues (S2 Fig). Immunologically, Mina KO mice exhibited normal frequencies of lymphoid and myeloid cell types in the thymus, spleen, peripheral (PLN) and mesenteric lymph nodes (MLN) (S1 Table). We did note that Mina KO mice exhibited a slight but significant decrease in MLN B cells and a significant increase in MLN CD8+ DCs. Frequencies of B cells and CD8+ DCs were normal in PLN and spleen. In order to further ascertain if the small difference in frequency of B cells and DC might contribute to a difference in immune response, we looked at the total cellularity of mesenteric lymph node and the total B cells and DC in the of Mina WT and KO mice upon TM infection. There was no difference in the cellularity of B and DC numbers upon TM infection. (S3 Fig). Additionally, in response to mitogenic stimulation T cells and B cells from Mina KO and WT control mice exhibited similar proliferative responses (S4 Fig). Taken together, these data indicated that Mina was required neither for ontogeny, fertility nor for immune system development. Thus, Mina KO mice appeared to be a suitable model with which to study Mina’s role in the intact host response to parasitic nematode infection.

### Mina deficient mice exhibit accelerated nematode expulsion

**Fig 1:**
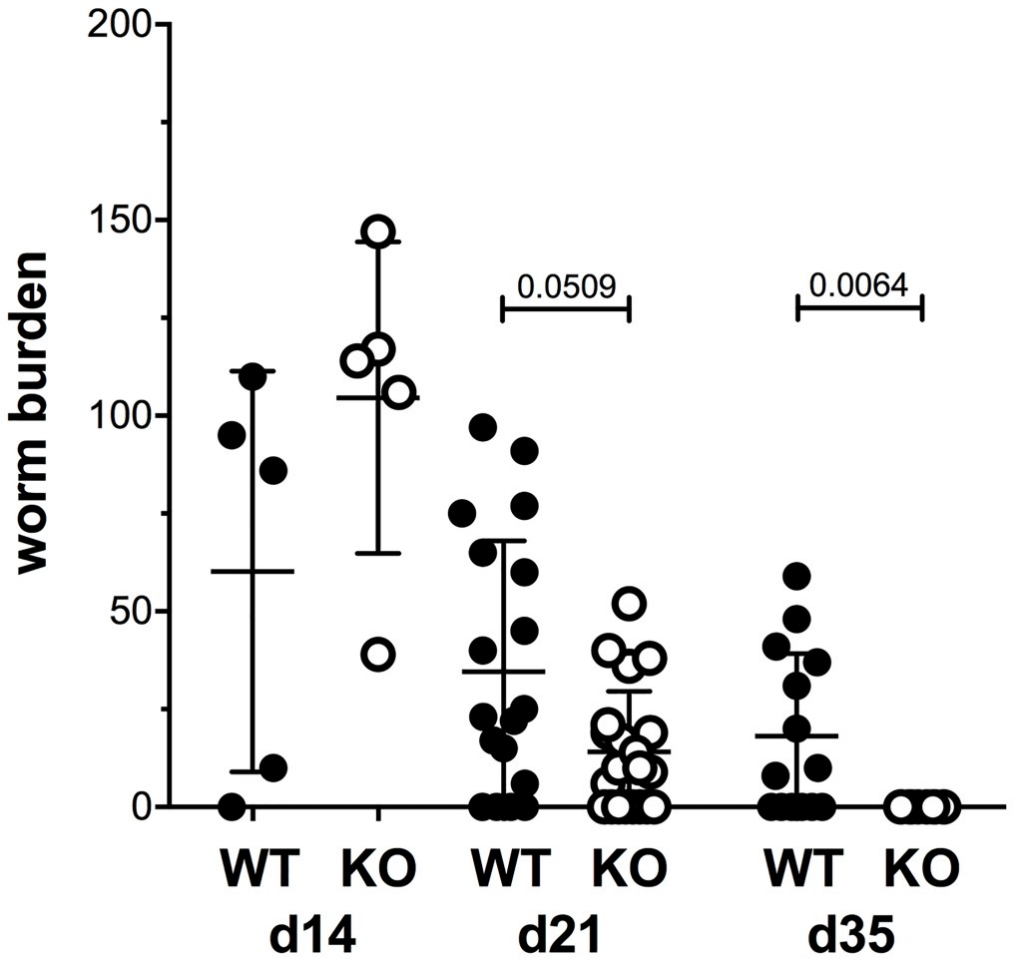
Accelerated clearance of TM in the absence of Mina. WT and Mina KO mice were infected by orogastric gavage with 150 TM embryonated eggs. At days 14, 21 and 35 dpi mice were sacrificed and ceca were collected. The ceca were dissected and resident larvae and adult worms were counted using a dissection microscope. Data are mean ± SEM (day 14 data from an experiment with n = 5; day 21 and 35 data from two independent experiments with WT: n = 19, KO: n = 21, d35; WT:n = 12, KO:n = 10). Statistical significance was computed by the nonparametric Mann-Whitney test.

Embryonated TM eggs were administered perorally to cohorts of Mina KO and WT littermate control mice and worm burden was measured over a 35-day infection course (Fig 1). Consistent with previous studies showing C57BL/6 mice to be moderately susceptible to TM [13], WT littermate control mice (N5B6) exhibited detectable parasite levels throughout the infection course; although, numbers dropped by ~70% between 14 and 35 days post infection (dpi). By contrast, parasites in Mina KO mice trended strongly downward by 21 dpi and were undetectable by 35 dpi, reflecting expulsion kinetics similar to resistant BALB/c strain mice [15]. Histological examination of the TM infected cecum sections suggested lesser inflammation in the KO mice compared to Mina WT at 21 and 35 dpi (S5 Fig). Thus, Mina is a non-redundant negative regulator of whipworm expulsion whose impairment releases the activity of a latent anthelmintic pathway.

### Mina deficient mice exhibit impaired parasite-specific Th1 but not Th2 responses

**Fig 2:**
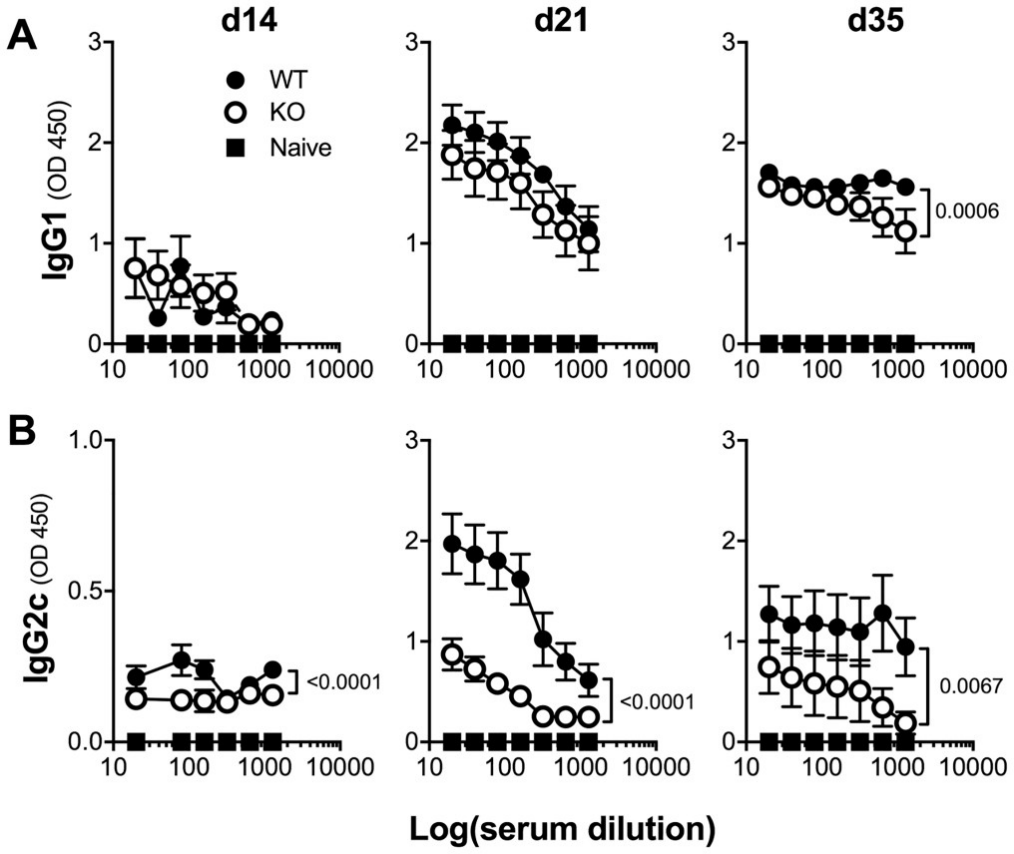
Normal TM-specific IgG1 and impaired IgG2c response in the absence of Mina. WT and Mina KO mice were infected by oral gavage with 150 TM embryonated eggs. Serum samples collected from infected WT or Mina KO mice were analyzed by ELISA on days 14, 21 and 35 dpi for TM antigen specific (**A**) IgG1 and (**B**) IgG2c. Data are mean ± SEM (d14, n = 6, d21, n = 11 each, d32 n = 6 each in Mina WT and KO respectively). OD260 curves were fitted using a 4 parameter logistic curve model and EC50s of fitted curves were compared to determine statistical significance and delta EC50.

Th2- and Th1-type host responses are known, respectively, to promote and antagonize expulsion of parasitic GI nematodes [13]. The Th2-type response, principally mediated by IL13, is critical for worm expulsion. This response stimulates ILC2 cells, triggering numerous effector pathways including increased epithelial cell turnover, goblet cell hyperplasia, mucus secretion, smooth muscle contractility and peristalsis [11, 16]. Thus, to investigate whether accelerated nematode expulsion in Mina KO mice was associated with altered Th1/Th2 balance, we measured levels of TM-specific serum IgG1 and IgG2c, hallmark Th2- and Th1-dependent antibody isotypes [17]. WT mice exhibited a low but detectable anti-TM IgG1 response at 14 dpi that peaked around 21 dpi and the response stayed similar at 35 dpi (Fig 2A). The response of Mina KO mice was similar to WT, apart from a slight decrease in titer by 35 dpi (~2 fold) that was consistent with accelerated parasite expulsion kinetics [18]. Total IgE levels at 0 and 21 dpi were also similar between Mina KO and WT littermates (S6 Fig). Together, these results indicated that Mina was dispensable for the Th2-type response to TM. This was further supported by finding normal levels of TSLP, IL33 and IL13 in day 21 TM infected Mina KO MLN (S7 Fig). Though unexpected in light of our previous work showing that Mina could repress *Il4* [12], this result was consistent with our finding of normal in vitro Th2 differentiation by Mina KO naïve CD4 T cells and suggested that Mina may be a redundant *Il4* regulator. In contrast to the relatively unperturbed Th2-type response to TM infection, the Th1-type response (reflected in serum IgG2c level) was dramatically impaired in the absence of Mina (Fig 2B). Thus, Mina is dispensable for the Th2- but essential for the Th1-type response to TM.

**Fig 3:**
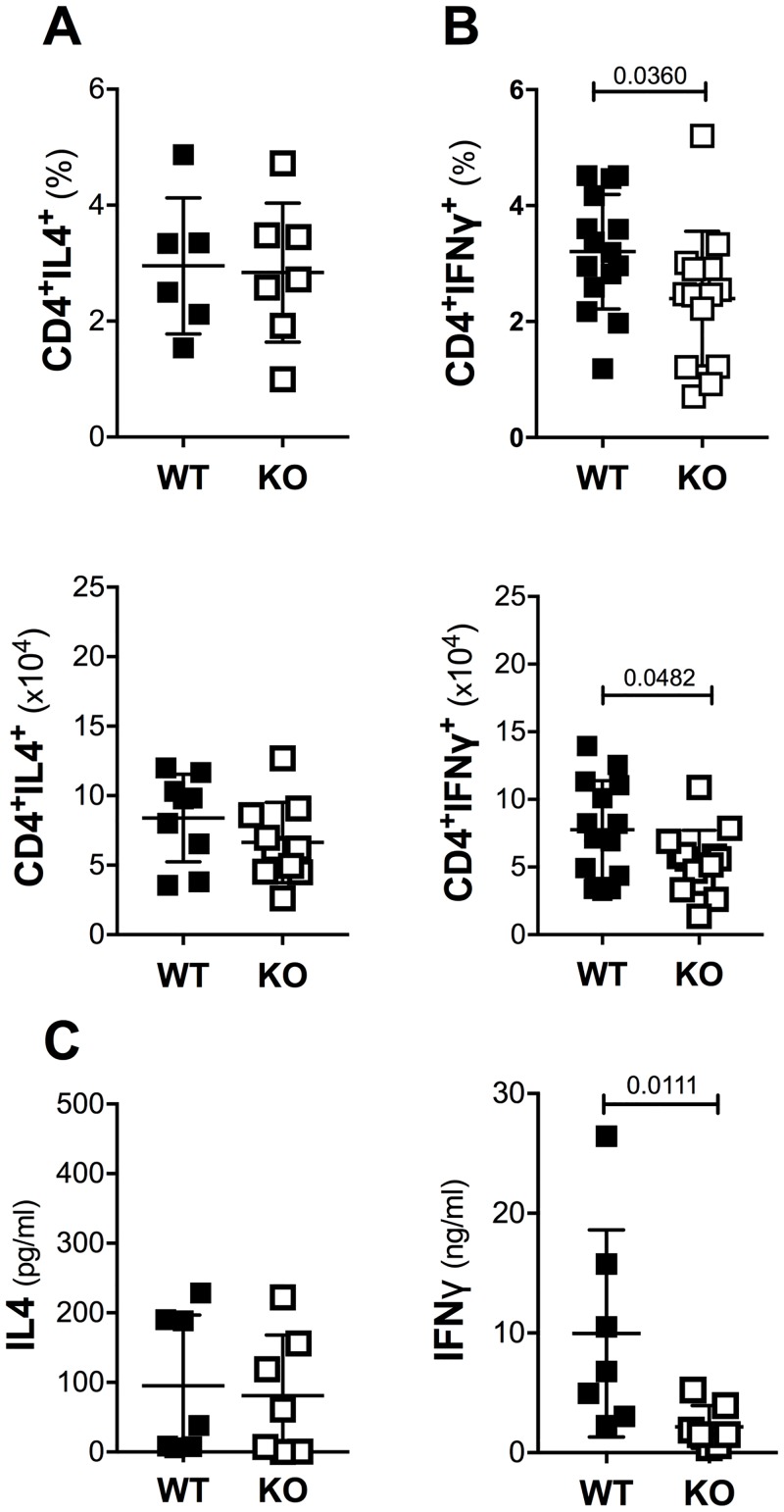
Th2 and Th1 response to TM in Mina KO mice. Shown are the percentage and absolute number of (A) CD4^+^IL4^+^ cells and (B) CD4^+^IFNγ^+^ cells and (C) the level of secreted IL4 and IFNγ from mesenteric lymph node cells of Mina KO and WT littermate control mice infected 21 days earlier with 150 embryonated TM eggs. Data are mean ± SD (A: WT n = 8, KO n = 10 each, B: WT and KO: n = 15 and 14 each respectively, C: WT and KO n = 7 each respectively) from 2 independent experiments). Statistical significance was computed by the two-tailed Student’s t-test.

To confirm the inference from serum isotype analysis of normal Th2- and impaired Th1-type responses in TM-infected Mina KO mice, we used FACS and ELISA to measure intracellular and secreted IL4 and IFNγ from PMA/ionomycin-stimulated CD4 T cells in draining mesenteric lymph nodes at 21 dpi. Consistent with the serum Ig analysis, Mina KO and littermate control WT mice exhibited similar numbers and proportions of Th2 cells as well as secreted IL4 (Fig 3). By contrast, Th1 cell number and percentage as well as secreted IFNγ levels were significantly lower in Mina KO than in WT (Fig 3). Together these results show that Mina is dispensable for Th2- but required to promote Th1-type responses to TM. Given the well-established antagonistic role of the Th1 response toward TM expulsion [13], these results suggest that Mina constrains TM expulsion, at least in part, by promoting the Th1 response.

### Mina acts in intestinal epithelial cells to constrain nematode expulsion and to promote a normal Th1-type response

**Fig 4:**
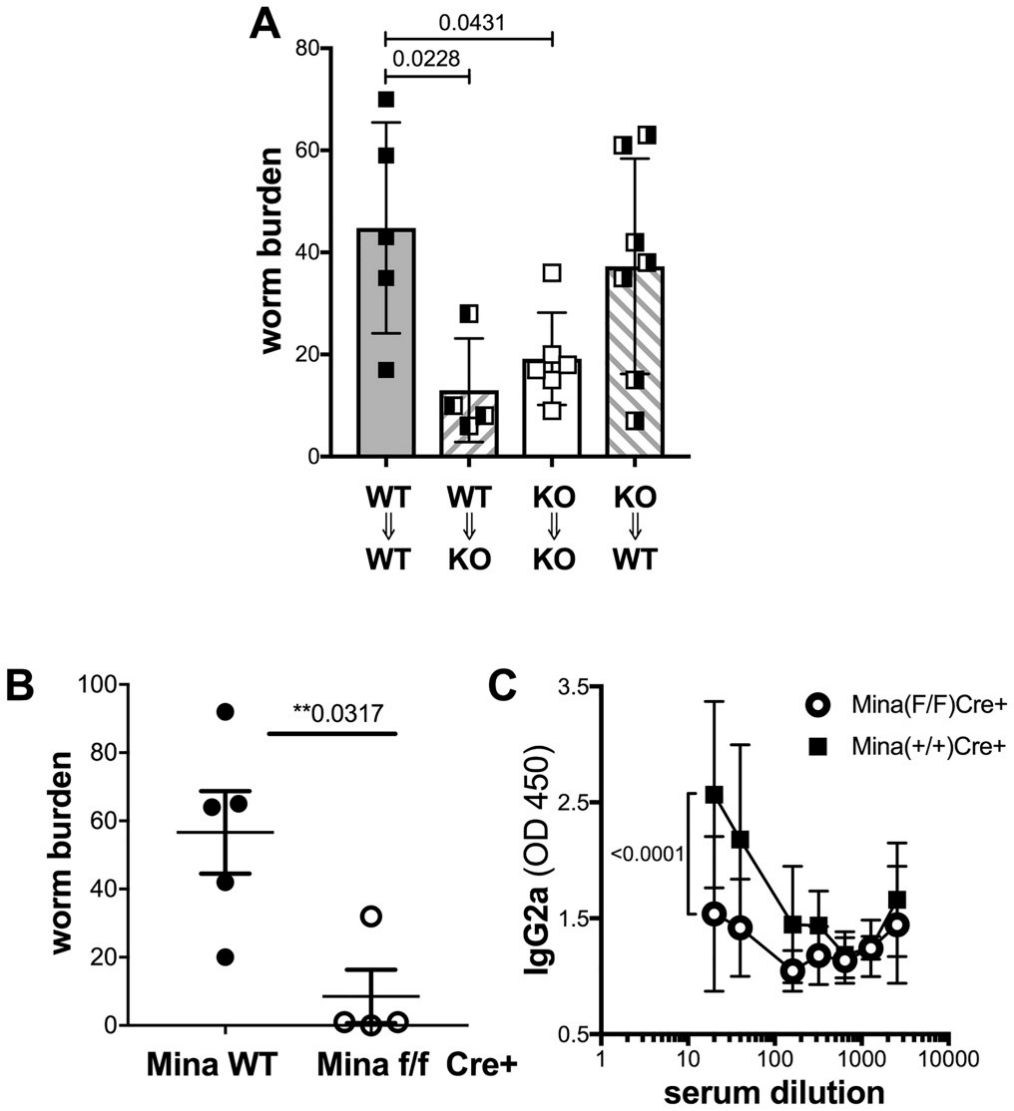
Worm burden and IgG2c response of TM-infected reciprocal bone chimeric and Mina^ΔIEC^ mice. Reciprocal bone marrow chimeras constructed as indicated (**A**) and Mina^ΔIEC^ mice (**B** and **C**) were infected with 150 TM eggs and d21 worm burden (**A** and **B**) and serum IgG2c response (**C**) were measured. Data are mean ± SD (bone marrow chimeras: n: WT to WT n = 5, WT to KO; n = 4, KO to KO n = 6, KO to WT n = 7 mice from 2 independent experiments; Mina^ΔIEC^: Mina +/+ Cre+ and Mina (fl/fl) Cre- n = 5, Mina Fl/Fl cre+ n = 4 mice from one of two independent experiments; IgG2c ELISA:WT n = 8, KO n = 6 from 2 independent experiments). For worm burden statistical significance was calculated by one way ANOVA; and for IgG2c level by fitting log (serum concentration) versus OD260 curves using a 4 parameter logistic curve model.

To examine Mina’s role in promoting Th1 development, Mina KO and WT naïve CD4 T cells were primed in Th1-inducing conditions and intracellular IFNγ was measured by FACS. Th1 differentiation proceeded normally in the absence of Mina (S8 Fig), suggesting that the attenuated Th1 response and, by inference, the accelerated parasite expulsion kinetics of TM-infected Mina KO mice arises from a T cell non-intrinsic defect. To identify the cellular compartment in which Mina acts to constrain parasite expulsion, we generated reciprocal bone marrow chimeras with WT and Mina KO mice. Autologously reconstituted Mina KO and WT chimeras recapitulated the TM expulsion phenotypes of their unmanipulated Mina KO and WT counterparts (Fig 4A), demonstrating that chimerism did not alter Mina’s role in parasite expulsion. Importantly, the phenotypes of heterologously reconstituted chimeras were dictated by host rather than hematopoietic donor genotypes. These results suggested that Mina acts in a non-hematopoietic compartment to retard TM expulsion.

We hypothesized that the non-hematopoietic source of Mina’s function (to retard worm expulsion) might be intestinal epithelial cells (IECs) proximal to where TM carries out its lifecycle [11]. To test this, we generated Mina^ΔIEC^ (Villin-Cre^+^::Mina^flox/flox^) and control (Villin-Cre^+^::Mina^+/+^ and Villin-Cre^-^::Mina^+/+^)) mice to model IEC-specific Mina ablation. Mina mRNA was readily detected in both control and Mina^ΔIEC^ splenocytes but only from control and not from Mina^ΔIEC^ IECs (S9 Fig). Thus, exon 3–4 deletion in Mina^ΔIEC^ mice was highly efficient and specifically targeted IECs. Next, Mina^ΔIEC^ and control mice were infected with TM and analysed at 21 dpi for worm burden (Fig 4B). Compared to control, Mina^ΔIEC^ mice exhibited significantly reduced worm burden, indicating that Mina expression in IECs was required to retard parasite expulsion. Similar results were observed when Mina KO mice were infected with Nippostrongylus brasiliensis NB (S10 Fig) suggesting that Mina deficiency results in accelerated worm expulsion. Importantly, compared to WT, Mina^ΔIEC^ mice also exhibited an impaired anti-TM serum IgG2c response (Fig 3C), indicating that a normal Th1-type response against TM also required IEC-intrinsic Mina activity.

### α-defensins are the major targets of Mina-mediated repression in TM infected IECs

**Fig 5:**
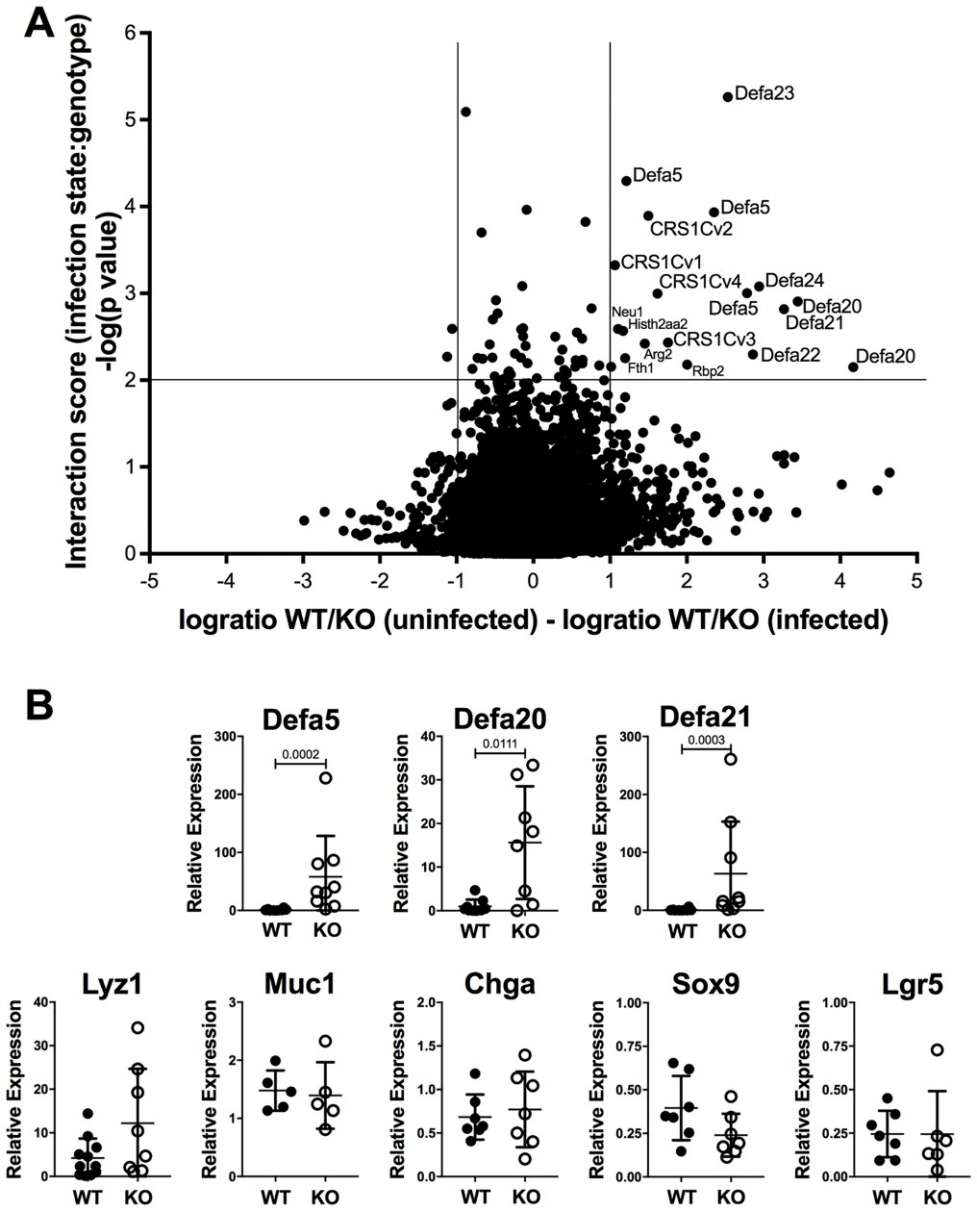
RNAseq expression profile of IECs. (**A**) Shown is a volcano plot depicting for each of 34,589 genes in IECs the magnitude of the mRNA expression effect size for the interaction between genotype and infection status parameters on the X axis and the statistical significance of each interaction on the Y axis. Red lines delineate the borders for |effect sizes| ≥ 2 fold and p ≤ 0.01. Genes in the top right quadrant represent those that are expressed at least 2 fold higher in Mina deficient infected IECs than in control uninfected ceca. Statistical analysis was carried out as described in the Methods. (B) Quantitative RT-PCR analysis of α-defensin (top row) and intestinal epithelial cell lineage marker genes (bottom row) in 21 dpi Mina KO and WT IECs. Data are mean ± SD (Def 20, 21, Criscv2 and v4; WT n = 11, KO n = 12 each, Defa1 WT n = 7, and KO n = 4, Defa5 WT n = 10, KO n = 9) (control genes: chga, Sox2, Lgr5 WT and KO n = 7 each, Muc1 n = 5 each, Lyz WT n = 12 and KO n = 8 resepctively from 2 independent experiments). Statistical significance was computed by the two-tailed Student’s t-test.

To explore the transcriptional basis of Mina’s IEC-intrinsic roles in TM expulsion and the Th1-type response, we performed RNAseq analysis of cecum and cecal IECs enriched from Mina-deficient and control mice, uninfected or 21 dpi. Strikingly, analysis of the data for genes whose differential expression required the interaction of genotype with infection state revealed a small but strong signature comprising 18 hits (Fig 5A). Remarkably 13 of 18 (72%) of these belonged to α-defensin or α-defensin-related gene families, known to be expressed in Paneth cells, secretory IECs that reside in small clusters at the base of the crypts of Lieberkühn (S2 Table) [19]. Paneth cells are normally found in the small intestine but can be induced ectopically in colonic crypts during inflammation such as that associated with nematode infection [20]. The 13 hits included 3 different copies of Defa5, 2 copies of defa20 and individual copies of Defa21, 22, 23 and 24 and 4 different cysteine-rich sequence (CRS) genes belonging to the CRS1C subfamily, encoding incompletely characterized α-defensin-related proteins [21]. Quantitative RT-PCR analysis of IECs from Mina KO and WT mice 21 dpi confirmed Mina-dependent repression of Defa5, 20 and 21 (Fig 5B) and one non-defensin gene, the Retinal binding protein 2 (Rbp2; S11 Fig). Importantly, Mina deficiency did not perturb expression of markers for five major IEC lineages (*Lyz1*, Paneth; *Muc1*, goblet; *Chga*, enteroendocrine; *Sox9*, tuft; and *Lgr5*, stem), indicating that TM did not perturb the normal distribution of gut epithelial cell lineages (Fig 5B). Together, these data suggest that Mina represses α-defensin mRNA levels directly rather than indirectly by impairing Paneth cell development or lifespan.

### α-defensins exhibit cytotoxic activity toward TM

**Fig 6:**
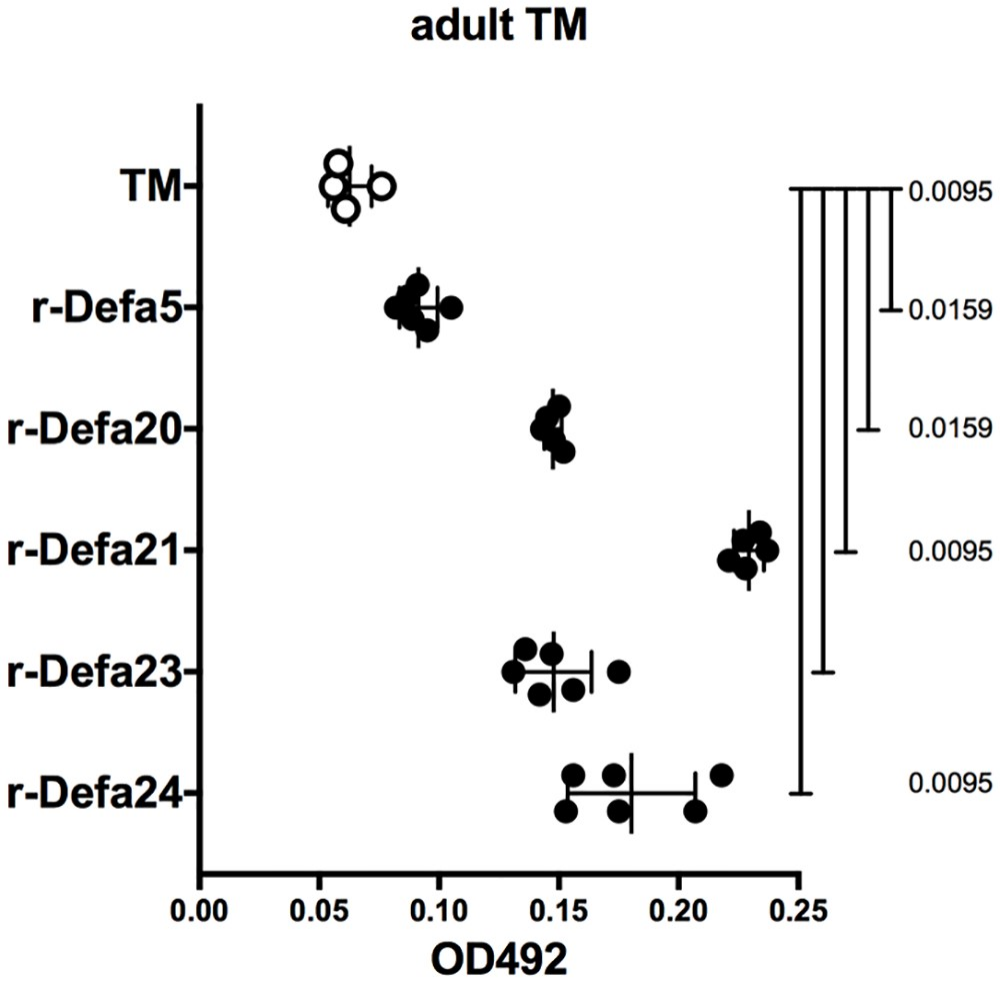
α-defensin cytotoxic activity toward adult TM. Cytotoxic activity of reduced α-defensins on adult TM males and females (obtained from female IL-4Rα KO mice on day 116 of infection) was measured by lactate dehydrogenase release assay. Data are mean ± SD (n = 6 each, and α -defensin 4: n = 5 adult worms) from 1 experiment. Statistical significance was computed by the Mann-Whitney test.

α-Defensins are 3–4 kDa highly cationic anti-microbial peptides that account for over 70% of the bactericidal activity of Paneth cell secretions and are important in providing both a first line of defense against microbial pathogens and in shaping commensal bacterial diversity [19] but have not been described to possess anthelmintic activity. We wondered whether they may act directly to kill or damage helminths. To test this idea, we incubated individual TM adults in microwells with reduced Defa5, 20, 21, 23 and 24 peptides for 1 hour before measuring lactate dehydrogenase release as a correlate of cytotoxicity. All five α-defensin isoforms exhibited significant cytotoxicity toward both male and female TM adults (Fig 6). Mina-regulated α-defensin isoforms were also active against L3 TM larvae (S12 Fig). Interestingly, cytotoxicity against L3 TM larvae was not restricted to Mina-regulated α-defensins. Defa1, 2, 4, 6 (not expressed in C57BL/6 [22] and Defa5 exhibited dose dependent cytotoxic activity against L3 TM larvae (S13 Fig). Curiously, cytotoxicity of reduced Defa1 and Defa4 was abolished by oxidation (S14 Fig). These in vitro results indicate that α-defensins possess cytotoxic activity toward TM and raise the possibility that α-defensins may possess cytotoxic activity toward helminths in vivo where they could contribute directly to accelerated worm expulsion. Additional work will be necessary to test this idea and to determine additionally whether derepressed α-defensins in TM infected Mina deficient mice might contribute to the impairment of the Th1 response.

## Discussion

Our previous work had led us to hypothesize that Mina, acting as an *Il4* repressor [12], would act to retard the highly Th2-dependent expulsion of parasitic helminths. Surprisingly however, analysis of in vitro Th2 differentiation in Mina KO cells revealed Mina not to be an essential regulator of Th2 development, perhaps due to functional redundancy with its close structural and evolutionary homolog NO66 [23]. Nevertheless, germline Mina deficiency did accelerate TM expulsion. Consistent with our in vitro data, accelerated expulsion was associated with a normal rather than an enhanced Th2 response. Providing a potential explanation for the accelerated expulsion phenotype, we discovered that Mina KO mice exhibited an impaired Th1 response to TM. As Th1 responses are well documented to promote chronic GI nematode infection [11], our results, point to a role for Mina in repressing worm expulsion by promoting the Th1 response.

However, as Mina KO T cells exhibited normal in vitro Th1 differentiation, we hypothesized that the impaired Th1 response and the accelerated expulsion phenotypes of Mina KO mice occurred via a T cell-extrinsic pathway. Indeed, reciprocal bone marrow chimera experiments demonstrated that accelerated worm expulsion required Mina deficiency in a non-hematopoietic compartment. TM challenge of Mina^ΔIEC^ mice (lacking Mina specifically in IECs) revealed a non-redundant IEC-intrinsic role for Mina in suppressing a latent anthelmintic pathway. In addition to accelerated TM expulsion, Mina^ΔIEC^ mice displayed an impaired serum IgG2c response, indicating an additional IEC-intrinsic role for Mina in promoting the anti-TM Th1-type response. As extended GI residence time can modulate the cytokine profile from Th2 to Th1 [18], the diminished Th1 response in infected Mina deficient mice could arise as a consequence (rather than being a causal component) of accelerated expulsion kinetics. However, as the IgG2c response in Mina KO mice was already impaired at 14 dpi while accelerated parasite expulsion did not become evident until 21 dpi, this explanation seems unlikely. Further, resistance can be conferred upon susceptible IL4 KO mice by neutralization of IFNγ [24]. Thus, Mina’s IEC-intrinsic role in repressing TM expulsion may derive, at least in part, from promotion of the Th1 response.

RNAseq analysis of ceca and cecal IECs from infected and uninfected Mina deficient and sufficient mice provided further insight into the molecular nature of the anthelmintic pathway negatively regulated by Mina. Analysis of the data to identify genes whose differential expression required interaction between genotype and infection status revealed a strikingly narrow perturbation signature focused on members of the rapidly evolving and widely distributed α-defensin gene family encoding anti-microbial peptides secreted by Paneth cells located at the base of the crypts of Lieberkuhn [20]. qRT-PCR analysis confirmed Mina- and TM-infection-dependent repression for 3 tested (of six) α-defensin genes and also for one (of two tested) non-α-defensin genes (encoding Retinal binding protein 2, Rbp2). Unperturbed expression of gene markers for the major IEC cell lineages, suggested that Mina acted directly to modulate target gene expression rather than indirectly by altering epithelial sublineage survival or proliferation.

Given the prominence of α-defensin and α-defensin-related genes in the Mina genetic perturbation signature we considered whether they might contribute to the IEC-intrinsic roles of Mina in response to TM infection. Upregulation of α-defensins in Mina^ΔIEC^ mice could promote accelerated TM expulsion via multiple non-mutually exclusive mechanisms. First, it is well established that α-defensins can shape commensal microbiota diversity [25]. Thus, α-defensins may act indirectly by altering commensal microbiota diversity, thereby eliminating niches essential for, or creating ones hostile toward, parasites [26]. Second, Paneth cell α-defensins have been reported to possess immunomodulatory activity. Reduced human HD5 can neutralize LPS and suppress macrophage production of TNF, suggesting a role as an LPS scavenger in the gut [27]. Further, Defa4 possesses anti-inflammatory activity on human blood leukocytes and human THP-1 monocytic cells in vitro and can ameliorate bacterial sepsis in vivo (personal communication from A. Ouellette). Thus, it is possible that α-defensins may repress the anti-TM Th1 response by altering the properties of dendritic cells that prime the Th1 response in the draining MLN. And finally, they may act directly to kill or damage helminths. In this regard, Rbp2, also negatively regulated by Mina in IECs, is another candidate for promotion of nematode expulsion by Th1 repression.

In the mouse, α-defensins are synthesized as inactive pro-peptides that during packaging into secretory granules are cleaved by matrix metalloproteinase-7 (MMP7) to generate functional carboxy-terminal α-defensin peptides liberated from acidic amino-terminal inhibitory prodomains [28, 29]. Mature α-defensin peptides are ~35 amino acid residues in length and exhibit a characteristic signature of six conserved cysteines that can oxidize to form three invariantly paired disulfide bonds, thereby creating a structure which in comparison to reduced α-defensin peptides is relatively resistant in vitro to proteolytic degradation [20, 30]. Exposure of adult and L3 larval TM to synthetic reduced mature α-defensin peptides lead to dose-dependent cytotoxicity, newly extending the target range of these anti-microbial proteins to metazoan organisms. In our experiments, the tested concentration of α-defensin peptides (50ug/ml) was on the high end in comparison to levels required in vitro to kill bacteria (1-10ug/ml) [22]; nevertheless, it was within the range estimated for α-defensin concentration at the point of release from Paneth cells (25–100 mg/ml) [20], at the ileal mucosal surface (90 to 450 ug/cm2) [31], in the adult ileal lumen (~250 ug/ml) [20] and within the crypt lumen (15–100 mg/ml) [19]. Further, cytotoxicity toward TM was resistant to 137 mM NaCl in PBS, a salt concentration that inhibited the microbicidal activity of certain α-defensins [19]. Interestingly, the microbicidal activity of α-defensin peptides is often greater when reduced as opposed to oxidized [32–36]. Similarly, cytotoxicity toward TM was observed with reduced but not oxidized peptides, raising the question whether such reduced peptides could escape proteolysis to function in vivo as cytotoxic effectors. A recent report revealed that the conserved cysteines in reduced α-defensins can chelate zinc and thereby adopt a protease resistant structure [33]. Further, full length functionally microbicidal α-defensins are detected in the colon even under reducing conditions [37]. Taken together with the unusually high levels of zinc found in Paneth cell secretory vesicles and the critical role of zinc in Paneth cell biology [38, 39], we hypothesize that protease-resistant, zinc-chelated α-defensin peptides may occur naturally, especially in the anaerobic reducing environment of the lower GI tract. Thus, the anthelminthic pathway held in check by Mina could be mediated, at least in part, by the direct cytotoxic activity of zinc-chelated α-defensins that may directly inflict damage upon or kill worms. Functional studies with zinc-chelated α-defensins, exogenous administration of α-defensin peptides and genetic perturbation experiments will be required to formally address the role of α-defensin peptides as direct anthelmintic effectors.

Mina is a 2-oxoglutarate-dependent oxygenase comprising a JmjC, a Winged Helix and a dimerization domain. It is highly enriched in the nucleolus and exists at lower levels in the nucleoplasm and cytoplasm [40]. To date, the only proven enzymatic target of Mina is ribosomal protein subunit Rpl27a which undergoes hydroxylation on histidine 39 [41]. The physiological ramifications of this posttranslational modification have yet to be determined and it is likely that Mina has additional targets. Mina is also reported to modulate specific positive regulatory histone marks, which may be consistent with a role as an epigenetic repressor [42, 43]. Indeed, our results suggest a role for Mina as a repressor of α-defensin mRNA. Importantly, IECs from Mina deficient mice exhibited unperturbed expression of gene markers for the major intestinal epithelial cell lineages, including Paneth cells that express α-defensins, thus ruling out an indirect role in the regulation of Paneth cell development or lifespan. Whether the role of Mina in α-defensin transcription is related to a direct role in epigenetic regulation, or more indirect effects on ribosome function or other pathway(s), remains to be elucidated. Additionally, we cannot rule out the possible role for goblet cells in expression of α-defensins in the Mina KO mice.

It is worth noting that the TM expulsion phenotype was stronger in Mina^ΔIEC^ as compared to germline Mina KO mice, reaching statistical significance on d21 in the former and requiring until d35 in the latter. Our previous work revealed a cell-intrinsic requirement for Mina in Th17 cell development [44]. Though the role of Th17 cells in TM expulsion remains to be elucidated, and a recent study has reported the failure of neutralizing antibodies to IL17 to influence expulsion kinetics [45], it is possible that an impaired Th17 response in Mina KO but not Mina^ΔIEC^ mice may have contributed to delayed expulsion in the former relative to the latter.

Finally, why evolve the Mina pathway if it acts to repress parasitic GI nematode expulsion? Self-regenerating 3D intestinal organoid cultures are a dynamic in vitro model of functional intact intestinal epithelium featuring villus- and crypt-like structures composed of the major IEC lineages [46]. TGFβ treatment of small intestinal organoid cultures induced *Mina* and repressed *α*-defensin gene expression (personal communication with Y. Eriguchi and A. Ouellette), consistent with a role for Mina in repressing *α-defensin* expression. As TGFβ is known to play a critical role in promoting chronic nematode infections [47], it is possible that activation of the Mina pathway by TGFβ [or a nematode TGFβ mimic [48]] is an evolved parasitic immune evasion mechanism. In this regard, it is notable that intron 1 of Mina contains a SMAD3-binding TGFβ response element (TRE) [49]. Further, the BALB/c but not the C57BL/6 allele of a SNP residing in the TRE abolished SMAD3 binding and TRE function, consistent with elevated Mina expression in C57BL/6 versus BALB/c cells [12] and correlating with the more rapid expulsion of TM from BALB/c as compared to C57BL/6 mice [13]. Thus, Mina activation may be at the epicenter of an evolutionary tug of war between host immunity and parasite immune evasion.

In summary, Mina represses a novel IEC-intrinsic anthelmintic pathway associated with a dampened Th1 response that may operate, at least in part, via mobilization of α-defensins. Further, α-defensins are newly revealed to possess direct cytotoxic activity toward parasitic GI nematodes, expanding the range of their pathogen targets to metazoans. Thus, Mina and its downstream effectors may be attractive targets for the development of new classes of anthelmintic drugs.

## Materials and methods

### Ethics statement

Mice were bred and maintained in specific pathogen-free conditions in accordance with the guidelines of the Institutional Animal Care and Use Committee of St. Jude Children’s Research Hospital, USA (Approved protocol number 453-100362-06/15) the UK Home Office Animals (Scientific Procedures) Act 1986 and Local Ethical Review Procedures (University of Oxford Medical Sciences Division Ethical Review Committee; Approved protocol number 30/2521); the Institutional Animal Care and Use Committee of Chiba University Japan (Approved protocol number 29–278), and the Institutional Animal Care and Use Committee of the Jikei University, Japan (Approved protocol number 2016–100).

Trichuris muris embryonated eggs were either generated in house as described in the methods or obtained from Dr David Artis or Dr Joesph Urban. The embyonated eggs were 6–7 weeks of age.

### Mice

Conditional Mina mutant mice were generated from gene targeted iTL BA1 (C57BL/6 x 129/SvEv) hybrid embryonic stem cells by InGenious Targeting Laboratory (Ronkonkoma, NY, USA) using the targeting vector shown in S1 Fig. Gene targeted founder mice were crossed with C57BL/6 Sox2-Cre+ to produce offspring harboring deletions spanning exons 3 and 4 and the neo cassette (Mina KO) or just the Neo cassette (Mina flox). Both Mina flox and Mina KO mice were backcrossed 5 times to C57BL/6 before use. Mina KO and WT littermate control mice were obtained from heterozygous intercrosses. Villin-Cre+::Mina(fl/fl), Villin-Cre-::Mina(fl/fl) and Villin-Cre+::Mina(+/+) littermates were obtained by crossing Villin-Cre+::Mina(fl/+) and Villin-Cre-::Mina(fl/+).

### Generation of bone marrow chimeras

Bone marrow was extruded from femurs and tibias as described (Holst et al., 2006) and then depleted of CD4+ T cells by MACS (Miltenyi) as per manufacturer’s instructions. Cells were incubated with anti-CD4 antibody, washed and then passed through an autoMACS (Miltenyi). Negatively selected cells were washed, counted and used for injections. 5 x10^6^ CD4 T cell-depleted bone marrow cells were injected in the tail vein of irradiated (1100 rads) recipient mice. Chimeras were given at least 6 weeks for hematopoietic reconstitution prior to TM infection.

### Cytokines and antibodies

Cytokines and antibodies were obtained from eBioscience, BD biosciences and BioLegend, Thermofisher and Promega as indicated in S3 Table.

### Immunophenotyping

Cells were recovered from thymus, spleen, peripheral lymph nodes (inguinal, brachial and axillary and superficial cervical) and mesenteric lymph nodes (MLN). After staining with LIVE/DEAD Aqua, cells were blocked with anti-CD16/32 antibody for 5 min on ice and then stained with lymphoid (anti-CD4, anti-CD8, anti-B220) or myeloid (anti-CD11b, anti-CD11c, anti-CD8, anti-Gr1, anti-B220 and anti F4/80) antibody cocktails on ice for 30 minutes. Cells were analyzed by BD LSR Fortessa flow cytometer.

### Generation of TM E/S (excretory/secretory) antigens and embryonated eggs

TM E/S antigen and embryonated eggs were generated as described [50]. Briefly, 35 days following orogastric gavage of susceptible AKR/J mice with 300 embryonated TM eggs, adult worms were recovered from the cecum and large intestine. To induce excretion/secretion of antigens and eggs, worms were incubated at 37°C in RPMI medium for 60 minutes and transferred to fresh media for an additional 60 minutes. Worm-conditioned media was combined and centrifuged at 1500 rpm for 10 minutes and E/S antigen-containing supernatant and pelleted eggs recovered. To induce embryonation eggs were washed twice in water and maintained in water in the dark at room temperature for 6–7 weeks.

### Nematode infections

One hundred and fifty embryonated TM eggs administered by orogastric gavage. At various times post infection resident worms were counted in the cecum and proximal colon using a dissection microscope, as described previously [50]. Harvested ceca and proximal colons were flushed with PBS, fixed in 10% neutral-buffered formalin, embedded in paraffin, sectioned and H&E stained. Slides were scored in a blinded manner for severity of inflammation (1 = minimal, 2 = mild, 3 = moderate, 4 = marked, 5 = severe), ulceration (0 = normal, 1 = mild, 2 = moderate, 3 = severe), hyperplasia (0 = normal, 1 = mild, 2 = moderate, 3 = severe) and area of involvement (0 = normal, 1 = minimal, 2 = mild, 3 = moderate, 4 = severe). Total inflammation score was calculated as (inflammation + ulceration + hyperplasia) X extent.

For Nippostrongylus brasiliensis infection, Mina WT and KO mice were infected by subcutaneous injection with 500 L3 *Nippostrongylus brasiliensis* larvae. At 7 d.p.i. mice were sacrificed and intestines were collected, opened longitudinally and placed in warm water in a 37C water bath. After 2 hours, the worms that have migrated to the water are counted under a dissection microscope.

### Intracellular cytokine analysis

Cell suspensions were restimulated with either PMA and ionomycin (Sigma) for 5 hrs. Following incubation with Golgistop (BD biosciences) for the last 4 hrs of restimulation, cells were washed and sequentially stained with Live/Dead Fixable Aqua (Invitrogen) and then antibodies targeting cell surface antigens, before being fixed and permeabilized with Fixation/Permeabilization reagent (BD biosciences) and then stained for intracellular cytokines in Fixation/Permeabilization buffer (BD biosciences) according to manufacturer’s recommendations. Data from stained cells was collected on an LSRII (BD), Fortessa (BD) or Canto II (BD) and analysed using Flowjo software.

### Enzyme linked immunosorbent assay (ELISA) and Milliplex assay

TM E/S antigen-specific serum IgG1 and IgG2c ELISAs were carried out as described [15]. Briefly, serum was added to ELISA plates that had been coated overnight with TM E/S antigen (5ug/ml), washed and blocked in 5% fetal calf serum. Following a 2h 37°C incubation, plates were sequentially washed, incubated for 1 hour at room temperature with secondary antibodies (BD Biosciences: biotinylated anti-mouse IgG1, clone A85-1 or biotinylated anti-mouse IgG2c, clone R19-15), washed and incubated with streptavidin HRP (BD Pharmingen) for 30 minutes at room temperature and finally detected with TMB substrate (eBioscience). Enzymatic reactions were stopped with 1N H_2_SO_4_ and OD450 measured on a Spectramax (Molecular Devices) spectrophotometer.

IgE ELISA was carried out by using the IgE ELISA kit from Thermofisher according to manufacturer’s instructions. OD values were measured using a Spectrophotometer (Versamax).

IFNγ ELISA was carried out with the Mouse IFN gamma ELISA Ready-SET-Go! Kit (eBioscience, cat #88–7314) according to manufacturer’s instructions. TSLP (Mouse TSLP Quantikine ELISA kit, R&D systems) and IL13, IL10 and IL4 were measured using a Milliplex Map kit (Millipore) according to manufacturer’s instructions.

### Intestinal epithelial cell enrichment

Ceca were harvested, washed in PBS containing 0.5% BSA, penicillin and streptomycin (50ug/ml) to remove fecal contents and luminal sides exposed and incubated for 30 mins at 4°C in E-RPMI (5% FCS, 5mM EDTA, 0.5mM DTT) with DNase I (20ug/ml). Luminal mucosa was recovered by scraping with a glass slide, collected into 50 ml tubes and pipetted repeatedly to disperse cellular aggregates. After allowing large aggregates to settle, the overlying cecal epithelial cell-enriched suspension was recovered. Purity was routinely 70–80% CD45-EPCAM+, as confirmed by staining with Live/Dead Fixable Aqua (Invitrogen), anti-mouse CD45 APC (30-F11, eBioscience) and anti-mouse EPCAM PE (G8.8, eBioscience).

### Isolation of RNA from tissues and quantitative RT-PCR

Intact ceca or enriched cecal epithelial cells were collected in Qiazol (Qiagen), and homogenized using a Tissue Lyzer II (Qiagen). Total RNA was extracted using the RNA Microarray Kit (Qiagen) and quantified using a Nanodrop spectrophotometer (Thermo Fisher Scientific). cDNA was synthesized from total RNA using the High Capacity cDNA Synthesis Kit (Thermo Fisher Scientific) according to manufacturer’s instructions. Quantitative PCR was carried out using Taqman primers (Thermo Fisher Scientific) on a 7900HT qPCR instrument (Applied Biosystems/Thermo Fisher Scientific). The mRNA expression of each gene tested were normalized to naïve WT samples and assessed using relative quantification method. GAPDH was used as an endogenous control.

### RNAseq and bioinformatics analysis

Total RNA was isolated using QIAzol and the RNeasy Mini Kit (Qiagen) from enriched cecal epithelial cells of uninfected Villin-Cre+::Mina(flox/flox) and Villin-Cre+::Mina(+/+) littermates; and from ceca of Mina(-/-) and Mina(+/+) littermates 21 days following TM infection. RNA quality was assessed using an Agilent Bioanalyzer. Libraries were prepared from ~500 ng total RNA with the TruSeq RNA Library Preparation Kit v2 according to manufacturer’s instructions (Illumina, San Diego, CA). Paired end 100 cycle sequencing was performed on HiSeq 2000 or HiSeq 2500 sequencers according to manufacturer’s instructions (Illumina). FASTQ sequences were mapped to the mouse mm9 genome by a pipeline that serially employs STAR [51] and BWA [52]. Mapped reads were counted, gene level FPKM values were computed, and resulting data was log2 transformed with an in-house pipeline. Mapping statistics and PCA were used for quality control. A two-way ANOVA model with an interaction term was applied to each gene to statistically analyze genotype, treatment and the conditional interaction of these factors. Graphic visualizations were performed using Partek Genomics Suite 6.6 (St Louis, MO USA) and STATA MP/11.2 (College Station, TX USA).

### α-defensin preparation

Synthetic reduced (r-)Defa1, r-Defa2, r-Defa4, r-Defa5 and r-Defa6, r-Defa20, r-Defa21, r-Defa23 and r-Defa24 were purchased from Medical and Biological Laboratories Co., Ltd. (Nagoya, japan) and stored at -30°C until use. Three pairs of disulfide bonds were introduced into synthetic Defa1 and Defa4 (Medical and Biological Laboratories Co., Ltd., Nagoya, japan) by air oxidation as described previously [35]. Using reverse-phase high-performance liquid chromatography (RP-HPLC) reaction mixtures were applied to C-18 columns (SepaxHP-C18, 4.6 x 150mm, 5ml; Sepax Technologies, Newark, DE, USA) in 0.1% trifluoroacetic acid with an 18–36% acetonitrile gradient developed over 30min at 1ml/min. Purified oxidized (o-)Defa1 and o-Defa4 were lyophilized and stored at -30°C until use. Disulfide bond status of Defas was evaluated by acid-urea PAGE and MALDI-TOF MS (Voyager-DE PRO; Applied Biosystems, Carlsbad, Calif., USA) as described previously [36].

### α-defensin cytotoxicity assay

TM adult and L3 larvae were recovered 116 and 21–25 d.p.i, respectively, from ceca and proximal colons of male IL4RaKO [BALB/c-IL4R, Line # 4177; [53]]. 1–5 L3 larvae/well and 1 adult, respectively, were incubated with α-defensin peptides (5–50 ug/ml) in 100ul PBS at 37°C for 1hr prior to the addition of 100ul of LDH-cytotoxicity assay kit solution (BioVision catalog # K311-400, CA, USA) and OD492 measurement.

### Cell proliferation assays

CD4^+^T cells (CD4^+^CD25^−^CD45RB^hi^) and B cells (CD19^+^) from spleens and lymph nodes of Mina KO or WT litter mate control mice were FACS sorted on a Reflection (i-Cyt, Champaign, IL) following staining with anti-mouse CD4, anti-mouse CD45RB and anti-mouse CD25 (eBioscience) for T cells and anti-mouse CD19 for B cells. CD4^+^T cells (1x10^5^ cells/ml) were stimulated for 72h with soluble anti-CD28 (1ug/ml, eBioscience) in 96 well flat bottom plates coated with graded amounts of anti-CD3 (eBioscience). B cells (1x10^6^ cells/ml) were stimulated in 96 well flat bottom plates with graded amounts of LPS (O111:B4, Sigma) for 72 h. Cultures were pulsed with 1 μCi of [^3^H]-thymidine for the final 8h of assay and harvested with a Packard harvester. The counts per minute (cpm) were determined using a Packard Matrix 96 direct counter (Packard Biosciences).

### T cell differentiation

Purified CD4+T cells (CD4^+^CD25^−^CD45RB^hi^) from spleens and lymph nodes of Mina KO or WT litter mate control mice were FACS sorted on a Reflection (i-Cyt, Champaign, IL) following staining with anti-mouse CD4, anti-mouse CD45RB and anti-mouse CD25 (eBioscience) were differentiated in to Th1 or Th2 as per previously published conditions [54]. Briefly, CD4+T cells were stimulated in anti-CD3 coated plates (5ug/ml), anti-CD28 (1ug/ml) in presence of rIL12 (20ng/ml) and anti-IL4(10ng/ml) for Th1 and anti-IFNγ (10ng/ml), rIL4 (100ng/ml) and anti-IL12 (10ng/ml) for Th2 differentiation. Cells were cultured for three days and restimulated with PMA (50 ng/ml) and ionomycin (1 μg/ml) in presence of monensin to test for intracellular cytokine IFNγ and IL4.

### Immunoblots

Organs and tissues from Mina WT (+/+) and KO (-/-) mice were homogenized in UREA/SDS buffer and 100 μg protein extract resolved on large 10% SDS-PAGE electrophoresis gels before immunblotting with rabbit polyclonal antibodies against Mina (Invitrogen, cat. No. 40–9500) and FIH (Novus, cat. No. NB100-428).

### Statistical analysis

GraphPad Prism software (version 7.0; GraphPad Software Inc.) was used to perform statistical analyses as indicated in figure legends. Sample sizes were chosen on the basis of previous experience in the laboratory with respect to inherent variability. Outlier data was identified and excluded by ROUT (Q = 1%). Animals within each cohort were randomly assigned to treatment groups. Blinded analysis was not performed in these studies. For statistical analysis of IgG2c ELISA data, EC50 was used to compare the entire values in the data to assess differences in the linear portion of the curve.

## Supporting information

S1 Fig Structure, expression and function of gene-targeted null and floxed Mina alleles. (A) Structure of the targeting construct and WT, Floxed and KO Mina alleles. (B) Diagnostic PCR bands corresponding to WT (+), null (-) and floxed (fl) Mina alleles in mice with indicated genotypes. (C) Mina protein expression in organs and tissues from Mina WT (+/+) and KO (-/-) mice. Equal protein loading was verified by immunoblotting with an antibody raised against a closely-related enzyme, FIH.
(PDF)

S2 Fig qRT-PCR analysis of mina expression in Mina KO. RNA expression of Mina was assessed directly from uninfected and infected tissues of Mina WT and KO mice. Data are from uninfected WT and KO n = 7 each, and infected WT and KO, n = 6 each respectively.
(PDF)

S3 Fig Cellularity of TM infected Mina WT and KO. The total cellularity of TM infected Mina WT and KO were assessed. The total numbers of CD19+ and CD11c+DC are assessed by flow cytometry and plotted. Statistical analysis was carried out by Mann-Whitney test. There were no significant difference between the groups (WT and KO, n = 5 each for total cellularity and CD11cDC and for CD19+ cells WT n = 5; KO n = 3).
(PDF)

S4 Fig Proliferation of Mina KO T and B cells. CD4^+^CD25^−^CD45RB^hi^ T cells (A and B) and CD19^+^ B cells (C and D) isolated from combined lymph node and spleen of Mina KO or WT littermate control mice were stimulated, respectively, with plate-bound anti-CD3/soluble anti-CD28, CD3/CD28 Dynabeads, LPS and anti-IgM respectively. Data are mean ± SEM (n = 6 mice). Log(mitogen concentration) versus cpm curves were fitted using a 4 parameter logistic curve model and EC50s of fitted curves were compared to determine statistical significance.
(PDF)

S5 Fig Histology of cecum from TM infected Mina WT and KO mice. Cecum from uninfected and Trichuris muris infected WT and Mina KO mice were harvested at d21 post infection. (A)The tissues were assessed for inflammation severity by hematoxylin eosin staining as described in the methods and (B) data from the histological assessment is shown. Data are from two independent experiments (WT and KO n = 13).
(PDF)

S6 Fig Serum IgE response to TM in Mina KO mice. Total serum IgE level from naive and TM infected WT and Mina KO mice at d21 p.i. The mice were infected by orogastric gavage with 150 TM embryonated eggs. Data are mean ± SD (Naïve WT n = 6, and KO n = 4, TM infected WT n = 9, and KO n = 13 mice) from 2 independent experiments). Statistical significance was computed by the two-tailed Student’s t-test.
(PDF)

S7 Fig Th2 and Th17 response to TM in Mina KO mice. (A) Quantitative RT-PCR analysis of TSLP and IL33 mRNA in IECs from Mina KO and WT 21 dpi. Data are mean ± SD of n = 6 each of WT and KO for TSLP and WT n = 10, KO = 9 mice for IL33 from 2 independent experiments. TSLP ELISA was carried out on TM antigen stimulated mesenteric LN culture supernatants using a TSLP ELISA kit as described in methods. Data is from one of two independent experiments. (C) Cytokine analysis was performed for assessing IL17, IL13, IL4, IL10 using Milliplex cytokine analysis kit. Data are mean ± SEM (IL17; WT n = 8 and KO n = 10, IL13, WT n = 7 and KO n = 8, IL4; WT n = 9 and KO n = 7; IL10; WT n = 9 and KO = 10 mice; from 2 independent experiments). Statistical significance was computed by the two-tailed Student’s t-test.
(PDF)

S8 Fig In vitro differentiation of Mina KO CD4 T cells. Mina KO and WT littermate controls were differentiated under Th1 and Th2 conditions as described in methods. Shown are the mean ± SD (n = 3 mice for Th1 and n = 4 for Th2 from 1 of 2 representative experiments). Statistical significance was computed by the two-tailed Student’s t-test.
(PDF)

S9 Fig Splenic and IEC Mina expression in Mina^ΔIEC^ mice. Mina mRNA expression level in splenocytes and IECs from Mina^ΔIEC^ [VillinCre+::Mina(fl/fl)] and controls VillinCre+::Mina(+/+) and VillinCre-::Mina(fl/fl)] mice. Shown are the mean ± SEM (n = 11, 5 and 9 for Splenocytes and n = 7, 5, 8 for IECs respectively from 2 independent experiments). Statistical significance was computed by the two-tailed Student’s t-test.
(PDF)

S10 Fig Accelerated clearance of *Nippostrongylus brasiliensis* in the absence of Mina53. WT and Mina KO mice were infected by subcutaneous injection with 500 L3 *Nippostrongylus brasiliensis* larvae. At 7 dpi the mice were sacrificed and intestines were collected, dissected and resident larvae and adult worms were counted using a dissection microscope. Data are mean ± SD (n = 6 mice from one experiment). Statistical significance was computed by the Mann-Whitney test.
(PDF)

S11 Fig Quantitative RT-PCR analysis of Rbp2. Quantitative RT-PCR analysis of Rbp2 in 21 d.p.i. Mina KO and WT IECs. Data are mean ± SD (WT n = 8, and KO n = 5 mice from 2 independent experiments). Statistical significance was computed by the two-tailed Student’s t-test.
(PDF)

S12 Fig Cytotoxic activity of α-defensins toward L3 TM larvae. Cytotoxic activity of reduced (r-) α-defensins 5, 20, 21, 23 and 24 against L3 TM larvae was measured by lactate dehydrogenase release assay. Data are mean ± SD (n = 3 larvae), representative of 2–4 independent experiments. α-defensin 5 was positive in 3 of 4 experiments. Statistical significance was computed by two-tailed Student’s t-test.
(PDF)

S13 Fig Dose dependent cytotoxic activity of α-defensins toward L3 TM larvae. Cytotoxic activity of reduced (r-) α-defensins 1, 2, 4, 5 and 6 against L3 TM larvae was measured by lactate dehydrogenase release assay. Data are mean ± SD (n = 3 larvae) in 1 experiment. Statistical significance was computed by the two-tailed Student’s t-test.
(PDF)

S14 Fig Reduced but not oxidized α-defensins exhibit cytotoxic activity toward L3 TM larvae. Cytotoxic activity of reduced (r-) and oxidized (o-) α-defensins 1 and 4 against L3 TM larvae was measured by lactate dehydrogenase release assay. Data are mean ± SD (n = 3 larvae), representative of 2 independent experiments. Statistical significance was computed by the two-tailed Student’s t-test.
(PDF)

S1 Table Lymphoid and myeloid immunophenotypic analysis of Mina KO mice. Mina KO and WT littermate control mesenteric lymph node, peripheral lymph node, spleen and thymic cells stained for various cell surface and intracellular markers as described in the Material and Methods and analyzed for the percentage of different cell subsets. Data are mean ± SD (from 2 independent experiments). Statistical significance was computed by the Mann Whitney test.
(PDF)

S2 Table RNAseq genes with expression greater than 2-fold higher in infected Mina-deficient as compared to uninfected control IECs and with p < 0.01 and that were confirmed by qRT-PCR. Mature peptide sequences are in bold font.
(PDF)

S3 Table Cytokines and antibodies. (PDF)

S4 Table PCR primers and probes. (PDF)

## References

[1] de Silva NR, Brooker S, Hotez PJ, Montresor A, Engels D, Savioli L. Soil-transmitted helminth infections: updating the global picture. Trends in parasitology. 2003;19(12):547–51. . 14642761 10.1016/j.pt.2003.10.002

[2] Ihler CF. Anthelmintic resistance. An overview of the situation in the Nordic countries. Acta Vet Scand. 1995;52((Suppl 1)):S24.

[3] Geerts S, Gryseels B. Anthelmintic resistance in human helminths: a review. Trop Med Int Health. 2001;6(11):915–21. . 11703846 10.1046/j.1365-3156.2001.00774.x

[4] Vercruysse J, Albonico M, Behnke JM, Kotze AC, Prichard RK, McCarthy JS, et al Is anthelmintic resistance a concern for the control of human soil-transmitted helminths? Int J Parasitol Drugs Drug Resist. 2011;1(1):14–27. 10.1016/j.ijpddr.2011.09.002 . 24533260 PMC3913213

[5] Love S. Prevalence of anthelmintic resistance in sheep worms in Australia: a thumbnail sketch. Proccedings of the Australian sheep veterinarians conference, Barossa Valley, South Australia. 2011.

[6] Playford MC, Smith AN, Love S, Besier RB, Kluver P, Bailey JN. Prevalence and severity of anthelmintic resistance in ovine gastrointestinal nematodes in Australia (2009–2012). Aust Vet J. 2014;92(12):464–71. 10.1111/avj.12271 . 25424758

[7] Matthews JB. Anthelmintic resistance in equine nematodes. Int J Parasitol Drugs Drug Resist. 2014;4(3):310–5. 10.1016/j.ijpddr.2014.10.003 . 25516842 PMC4266799

[8] Gasbarre LC, Ballweber LR, Stromberg BE, Dargatz DA, Rodriguez JM, Kopral CA, et al Effectiveness of current anthelmintic treatment programs on reducing fecal egg counts in United States cow-calf operations. Can J Vet Res. 2015;79(4):296–302. . 26424910 PMC4581674

[9] Geurden T, Chartier C, Fanke J, di Regalbono AF, Traversa D, von Samson-Himmelstjerna G, et al Anthelmintic resistance to ivermectin and moxidectin in gastrointestinal nematodes of cattle in Europe. Int J Parasitol Drugs Drug Resist. 2015;5(3):163–71. 10.1016/j.ijpddr.2015.08.001 . 26448902 PMC4572401

[10] Roeber F, Jex AR, Gasser RB. Impact of gastrointestinal parasitic nematodes of sheep, and the role of advanced molecular tools for exploring epidemiology and drug resistance—an Australian perspective. Parasit Vectors. 2013;6:153 10.1186/1756-3305-6-153 . 23711194 PMC3679956

[11] Grencis RK. Immunity to helminths: resistance, regulation, and susceptibility to gastrointestinal nematodes. Annual review of immunology. 2015;33:201–25. 10.1146/annurev-immunol-032713-120218 . 25533702

[12] Okamoto M, Van Stry M, Chung L, Koyanagi M, Sun X, Suzuki Y, et al Mina, an Il4 repressor, controls T helper type 2 bias. Nat Immunol. 2009;10(8):872–9. 10.1038/ni.1747 . 19561615 PMC2825757

[13] Klementowicz JE, Travis MA, Grencis RK. Trichuris muris: a model of gastrointestinal parasite infection. Seminars in immunopathology. 2012;34(6):815–28. 10.1007/s00281-012-0348-2 . 23053395 PMC3496546

[14] Mori T, Okamoto K, Tanaka Y, Teye K, Umata T, Ohneda K, et al Ablation of Mina53 in mice reduces allergic response in the airways. Cell structure and function. 2013;38(2):155–67. . 23748603 10.1247/csf.13006

[15] Artis D, Wang ML, Keilbaugh SA, He W, Brenes M, Swain GP, et al RELMbeta/FIZZ2 is a goblet cell-specific immune-effector molecule in the gastrointestinal tract. Proc Natl Acad Sci U S A. 2004;101(37):13596–600. Epub 2004/09/02. 10.1073/pnas.0404034101 . 15340149 PMC518800

[16] von Moltke J, Ji M, Liang HE, Locksley RM. Tuft-cell-derived IL-25 regulates an intestinal ILC2-epithelial response circuit. Nature. 2016;529(7585):221–5. 10.1038/nature16161 . 26675736 PMC4830391

[17] Stevens TL, Bossie A, Sanders VM, Fernandez-Botran R, Coffman RL, Mosmann TR, et al Regulation of antibody isotype secretion by subsets of antigen-specific helper T cells. Nature. 1988;334(6179):255–8. 10.1038/334255a0 . 2456466

[18] Else KJ, Hultner L, Grencis RK. Modulation of cytokine production and response phenotypes in murine trichuriasis. Parasite Immunol. 1992;14(4):441–9. . 1437236 10.1111/j.1365-3024.1992.tb00018.x

[19] Ayabe T, Satchell DP, Wilson CL, Parks WC, Selsted ME, Ouellette AJ. Secretion of microbicidal alpha-defensins by intestinal Paneth cells in response to bacteria. Nat Immunol. 2000;1(2):113–8. 10.1038/77783 . 11248802

[20] Ouellette AJ. Paneth cell alpha-defensins in enteric innate immunity. Cell Mol Life Sci. 2011;68(13):2215–29. 10.1007/s00018-011-0714-6 . 21560070 PMC4073591

[21] Ouellette AJ, Lualdi JC. A novel mouse gene family coding for cationic, cysteine-rich peptides. Regulation in small intestine and cells of myeloid origin. J Biol Chem. 1990;265(17):9831–7. . 2351676

[22] Shanahan MT, Tanabe H, Ouellette AJ. Strain-specific polymorphisms in Paneth cell alpha-defensins of C57BL/6 mice and evidence of vestigial myeloid alpha-defensin pseudogenes. Infection and immunity. 2011;79(1):459–73. 10.1128/IAI.00996-10 . 21041494 PMC3019906

[23] Chowdhury R, Sekirnik R, Brissett NC, Krojer T, Ho CH, Ng SS, et al Ribosomal oxygenases are structurally conserved from prokaryotes to humans. Nature. 2014;510(7505):422–6. 10.1038/nature13263 . 24814345 PMC4066111

[24] Hepworth MR, Grencis RK. Disruption of Th2 immunity results in a gender-specific expansion of IL-13 producing accessory NK cells during helminth infection. J Immunol. 2009;183(6):3906–14. 10.4049/jimmunol.0900577 . 19692641 PMC2738657

[25] Salzman NH, Hung K, Haribhai D, Chu H, Karlsson-Sjoberg J, Amir E, et al Enteric defensins are essential regulators of intestinal microbial ecology. Nat Immunol. 2010;11(1):76–83. 10.1038/ni.1825 . 19855381 PMC2795796

[26] Hayes KS, Bancroft AJ, Goldrick M, Portsmouth C, Roberts IS, Grencis RK. Exploitation of the intestinal microflora by the parasitic nematode Trichuris muris. Science. 2010;328(5984):1391–4. 10.1126/science.1187703 . 20538949 PMC3428897

[27] Wang C, Shen M, Zhang N, Wang S, Xu Y, Chen S, et al Reduction Impairs the Antibacterial Activity but Benefits the LPS Neutralization Ability of Human Enteric Defensin 5. Scientific reports. 2016;6:22875 10.1038/srep22875 . 26960718 PMC4785407

[28] Wilson CL, Ouellette AJ, Satchell DP, Ayabe T, Lopez-Boado YS, Stratman JL, et al Regulation of intestinal alpha-defensin activation by the metalloproteinase matrilysin in innate host defense. Science. 1999;286(5437):113–7. . 10506557 10.1126/science.286.5437.113

[29] Shirafuji Y, Tanabe H, Satchell DP, Henschen-Edman A, Wilson CL, Ouellette AJ. Structural determinants of procryptdin recognition and cleavage by matrix metalloproteinase-7. J Biol Chem. 2003;278(10):7910–9. 10.1074/jbc.M210600200 . 12482850

[30] Selsted ME, Ouellette AJ. Mammalian defensins in the antimicrobial immune response. Nat Immunol. 2005;6(6):551–7. 10.1038/ni1206 . 15908936

[31] Salzman NH, Ghosh D, Huttner KM, Paterson Y, Bevins CL. Protection against enteric salmonellosis in transgenic mice expressing a human intestinal defensin. Nature. 2003;422(6931):522–6. 10.1038/nature01520 . 12660734

[32] Schroeder BO, Wu Z, Nuding S, Groscurth S, Marcinowski M, Beisner J, et al Reduction of disulphide bonds unmasks potent antimicrobial activity of human beta-defensin 1. Nature. 2011;469(7330):419–23. 10.1038/nature09674 . 21248850

[33] Zhang Y, Cougnon FB, Wanniarachchi YA, Hayden JA, Nolan EM. Reduction of human defensin 5 affords a high-affinity zinc-chelating peptide. ACS Chem Biol. 2013;8(9):1907–11. 10.1021/cb400340k . 23841778 PMC3783636

[34] Jaeger SU, Schroeder BO, Meyer-Hoffert U, Courth L, Fehr SN, Gersemann M, et al Cell-mediated reduction of human beta-defensin 1: a major role for mucosal thioredoxin. Mucosal Immunol. 2013;6(6):1179–90. 10.1038/mi.2013.17 . 23571504 PMC3806438

[35] Maemoto A, Qu X, Rosengren KJ, Tanabe H, Henschen-Edman A, Craik DJ, et al Functional analysis of the alpha-defensin disulfide array in mouse cryptdin-4. J Biol Chem. 2004;279(42):44188–96. 10.1074/jbc.M406154200 . 15297466

[36] Masuda K, Sakai N, Nakamura K, Yoshioka S, Ayabe T. Bactericidal activity of mouse alpha-defensin cryptdin-4 predominantly affects noncommensal bacteria. J Innate Immun. 2011;3(3):315–26. 10.1159/000322037 . 21099205

[37] Mastroianni JR, Ouellette AJ. Alpha-defensins in enteric innate immunity: functional Paneth cell alpha-defensins in mouse colonic lumen. J Biol Chem. 2009;284(41):27848–56. 10.1074/jbc.M109.050773 . 19687006 PMC2788835

[38] Dinsdale D. Ultrastructural localization of zinc and calcium within the granules of rat Paneth cells. J Histochem Cytochem. 1984;32(2):139–45. . 6693753 10.1177/32.2.6693753

[39] Giblin LJ, Chang CJ, Bentley AF, Frederickson C, Lippard SJ, Frederickson CJ. Zinc-secreting Paneth cells studied by ZP fluorescence. J Histochem Cytochem. 2006;54(3):311–6. 10.1369/jhc.5A6724.2005 . 16260591

[40] Eilbracht J, Kneissel S, Hofmann A, Schmidt-Zachmann MS. Protein NO52—a constitutive nucleolar component sharing high sequence homologies to protein NO66. Eur J Cell Biol. 2005;84(2–3):279–94. 10.1016/j.ejcb.2004.12.022 . 15819408

[41] Ge W, Wolf A, Feng T, Ho CH, Sekirnik R, Zayer A, et al Oxygenase-catalyzed ribosome hydroxylation occurs in prokaryotes and humans. Nat Chem Biol. 2012;8(12):960–2. 10.1038/nchembio.1093 . 23103944 PMC4972389

[42] Lu Y, Chang Q, Zhang Y, Beezhold K, Rojanasakul Y, Zhao H, et al Lung cancer-associated JmjC domain protein mdig suppresses formation of tri-methyl lysine 9 of histone H3. Cell Cycle. 2009;8(13):2101–9. 10.4161/cc.8.13.8927 . 19502796

[43] Huang MY, Xuan F, Liu W, Cui HJ. MINA controls proliferation and tumorigenesis of glioblastoma by epigenetically regulating cyclins and CDKs via H3K9me3 demethylation. Oncogene. 2017;36(3):387–96. 10.1038/onc.2016.208 . 27292258

[44] Yosef N, Shalek AK, Gaublomme JT, Jin H, Lee Y, Awasthi A, et al Dynamic regulatory network controlling TH17 cell differentiation. Nature. 2013;496(7446):461–8. 10.1038/nature11981 . 23467089 PMC3637864

[45] Grencis RK, Humphreys NE, Bancroft AJ. Immunity to gastrointestinal nematodes: mechanisms and myths. Immunological reviews. 2014;260(1):183–205. 10.1111/imr.12188 . 24942690 PMC4141702

[46] Sato T, Vries RG, Snippert HJ, van de Wetering M, Barker N, Stange DE, et al Single Lgr5 stem cells build crypt-villus structures in vitro without a mesenchymal niche. Nature. 2009;459(7244):262–5. 10.1038/nature07935 . 19329995

[47] Grainger JR, Smith KA, Hewitson JP, McSorley HJ, Harcus Y, Filbey KJ, et al Helminth secretions induce de novo T cell Foxp3 expression and regulatory function through the TGF-beta pathway. The Journal of Experimental Medicine. 2010;207(11):2331–41. Epub 2010/09/30. 10.1084/jem.20101074 . 20876311 PMC2964568

[48] Gomez-Escobar N, Gregory WF, Maizels RM. Identification of tgh-2, a filarial nematode homolog of Caenorhabditis elegans daf-7 and human transforming growth factor beta, expressed in microfilarial and adult stages of Brugia malayi. Infection and immunity. 2000;68(11):6402–10. . 11035752 10.1128/iai.68.11.6402-6410.2000 PMC97726

[49] Lian SL, Mihi B, Koyanagi M, Nakayama T, Bix M. A SNP uncoupling Mina expression from the TGFbeta signaling pathway. Immun Inflamm Dis. 2017 10.1002/iid3.191 . 28967702 PMC5818440

[50] Wakelin D. Acquired immunity to Trichuris muris in the albino laboratory mouse. Parasitology. 1967;57(3):515–24. . 6048569 10.1017/s0031182000072395

[51] Dobin A, Davis CA, Schlesinger F, Drenkow J, Zaleski C, Jha S, et al STAR: ultrafast universal RNA-seq aligner. Bioinformatics (Oxford, England). 2013;29(1):15–21. 10.1093/bioinformatics/bts635 . 23104886 PMC3530905

[52] Li H, Durbin R. Fast and accurate short read alignment with Burrows-Wheeler transform. Bioinformatics (Oxford, England). 2009;25(14):1754–60. 10.1093/bioinformatics/btp324 . 19451168 PMC2705234

[53] Noben-Trauth N, Shultz LD, Brombacher F, Urban JF Jr., Gu H, Paul WE. An interleukin 4 (IL-4)-independent pathway for CD4+ T cell IL-4 production is revealed in IL-4 receptor-deficient mice. Proc Natl Acad Sci U S A. 1997;94(20):10838–43. 9380721 10.1073/pnas.94.20.10838 PMC23501

[54] Li J. *In vitro* Differentiation of Mouse Th0, Th1 and Th2 from Naïve CD4 T Cells. *Bio-protocol* 2011; Bio101: e157 10.21769/BioProtoc.157

