# Supplementary material for: Myc-induced nuclear antigen constrains a latent intestinal epithelial cell-intrinsic anthelmintic pathway
Source: PLoS One. 2019 Feb 26;14(2):e0211244. doi: 10.1371/journal.pone.0211244 (PMC6391002; doi:10.1371/journal.pone.0211244)
Supplement: S1 Fig — (A) Structure of the targeting construct and WT, Floxed and KO Mina alleles. (B) Diagnostic PCR bands corresponding to WT (+), null (-) and floxed (fl) Mina alleles in mice with indicated genotypes. (C) Mina protein expression in organs and tissues from Mina WT (+/+) and KO (-/-) mice. Equal protein loading was verified by immunoblotting with an antibody raised against a closely-related enzyme, FIH. (PDF) [file pone.0211244.s001.pdf]

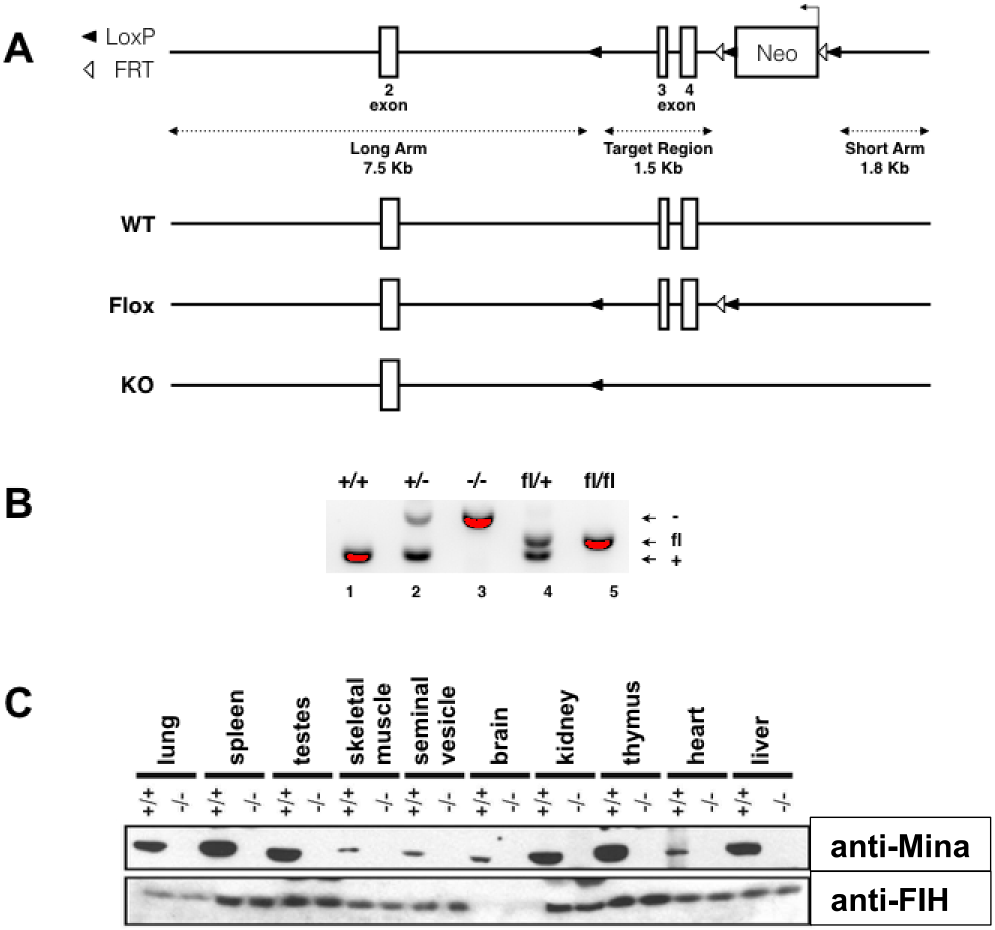

Fig S1. Structure, expression and function of gene-targeted null and floxed Mina alleles. (A) Structure of the targeting construct and WT, Floxed and KO Mina alleles. (B) Diagnostic PCR bands corresponding to WT (+), null (-) and floxed (fl) Mina alleles in mice with indicated genotypes. (C) Mina protein expression in organs and tissues from Mina WT (+/+) and KO (-/-) mice. Equal protein loading was verified by immunoblotting with an antibody raised against a closely-related enzyme, FIH.
