# Supplementary material for: Myc-induced nuclear antigen constrains a latent intestinal epithelial cell-intrinsic anthelmintic pathway
Source: PLoS One. 2019 Feb 26;14(2):e0211244. doi: 10.1371/journal.pone.0211244 (PMC6391002; doi:10.1371/journal.pone.0211244)
Supplement: S2 Fig — RNA expression of Mina was assessed directly from uninfected and infected tissues of Mina WT and KO mice. Data are from uninfected WT and KO n = 7 each, and infected WT and KO, n = 6 each respectively. (PDF) [file pone.0211244.s002.pdf]

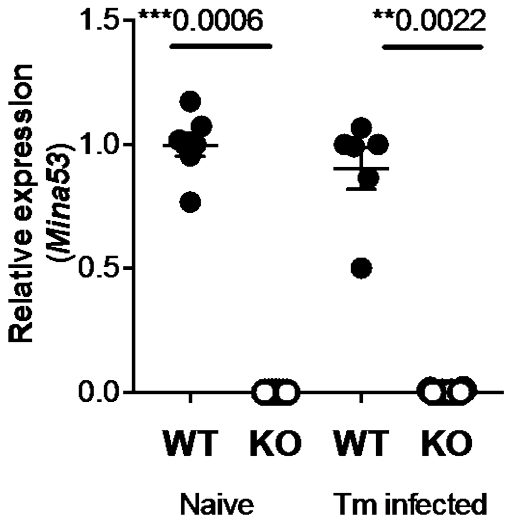


**Fig S2. Expression level of Mina in cecum tissue.** Gene expression of Mina was assessed in uninfected or infected Mina WT or KO by qRT_PCR using specific primers and probes. Uninfected WT and KO , n=7., Infected WT and KO n=6 respectively. Statistical significance was computed using the Mann-Whitney test.
