# Supplementary material for: Myc-induced nuclear antigen constrains a latent intestinal epithelial cell-intrinsic anthelmintic pathway
Source: PLoS One. 2019 Feb 26;14(2):e0211244. doi: 10.1371/journal.pone.0211244 (PMC6391002; doi:10.1371/journal.pone.0211244)
Supplement: S3 Fig — The total cellularity of TM infected Mina WT and KO were assessed. The total numbers of CD19+ and CD11c+DC are assessed by flow cytometry and plotted. Statistical analysis was carried out by Mann-Whitney test. There were no significant difference between the groups (WT and KO, n = 5 each for total cellularity and CD11cDC and for CD19+ cells WT n = 5; KO n = 3). (PDF) [file pone.0211244.s003.pdf]

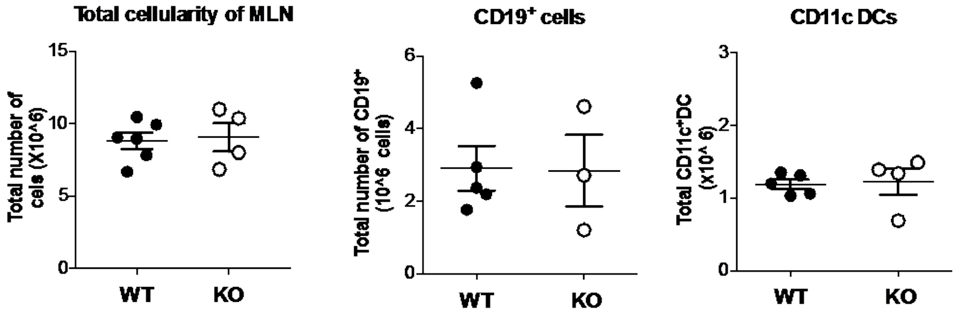

Fig S3. Cellularity of TM infected Mina WT and KO. The total cellularity, total number of $CD19^+$ and $CD11c^+$DC from MLN of TM infected Mina WT and KO were assessed by flow cytometry and plotted. Statistical analysis was carried out by Mann-Whitney test. (WT and KO, n=5 each for total cellularity and $CD11c^+$ DC and for $CD19^+$ cells WT n=5; KO n=3).
