# Supplementary material for: Myc-induced nuclear antigen constrains a latent intestinal epithelial cell-intrinsic anthelmintic pathway
Source: PLoS One. 2019 Feb 26;14(2):e0211244. doi: 10.1371/journal.pone.0211244 (PMC6391002; doi:10.1371/journal.pone.0211244)
Supplement: S4 Fig — CD4+CD25−CD45RBhi T cells (A and B) and CD19+ B cells (C and D) isolated from combined lymph node and spleen of Mina KO or WT littermate control mice were stimulated, respectively, with plate-bound anti-CD3/soluble anti-CD28, CD3/CD28 Dynabeads, LPS and anti-IgM respectively. Data are mean ± SEM (n = 6 mice). Log(mitogen concentration) versus cpm curves were fitted using a 4 parameter logistic curve model and EC50s of fitted curves were compared to determine statistical significance. (PDF) [file pone.0211244.s004.pdf]

D

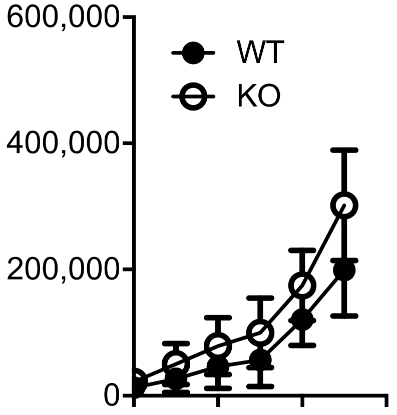

Fig S4. Proliferation of Mina KO T and B cells. CD4$^+$CD25$^-$CD45RB^{hi} T cells (A and B) and CD19$^+$ B cells (C and D) isolated from combined lymph node and spleen of Mina KO or WT littermate control mice were stimulated, respectively, with plate-bound anti-CD3/soluble anti-CD28, CD3/CD28 Dynabeads for A and B, LPS and anti-IgM for C and D respectively. Data are mean ± SEM (n = 6 mice). Log(mitogen concentration) versus cpm curves were fitted using a 4 parameter logistic curve model and EC50s of fitted curves were compared to determine statistical significance.
