# Supplementary material for: Myc-induced nuclear antigen constrains a latent intestinal epithelial cell-intrinsic anthelmintic pathway
Source: PLoS One. 2019 Feb 26;14(2):e0211244. doi: 10.1371/journal.pone.0211244 (PMC6391002; doi:10.1371/journal.pone.0211244)
Supplement: S5 Fig — Cecum from uninfected and Trichuris muris infected WT and Mina KO mice were harvested at d21 post infection. (A)The tissues were assessed for inflammation severity by hematoxylin eosin staining as described in the methods and (B) data from the histological assessment is shown. Data are from two independent experiments (WT and KO n = 13). (PDF) [file pone.0211244.s005.pdf]

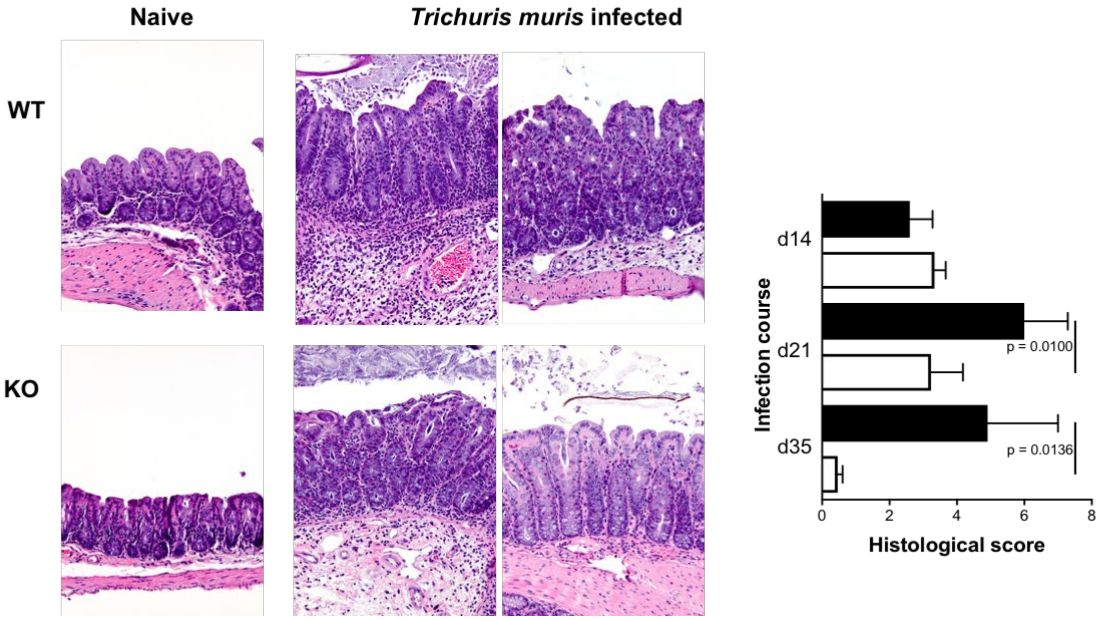

Fig S5. Histology of cecum from TM infected Mina WT and KO mice. Cecum from uninfected and Trichuris muris infected WT and Mina KO mice were harvested at d21 post infection. (A)The tissues were assessed for inflammation severity by hematoxylin eosin staining as described in the methods and (B) data from the histological assessment is shown. Data are from two independent experiments (WT and KO n=13).
