# Supplementary material for: Myc-induced nuclear antigen constrains a latent intestinal epithelial cell-intrinsic anthelmintic pathway
Source: PLoS One. 2019 Feb 26;14(2):e0211244. doi: 10.1371/journal.pone.0211244 (PMC6391002; doi:10.1371/journal.pone.0211244)
Supplement: S6 Fig — Total serum IgE level from naive and TM infected WT and Mina KO mice at d21 p.i. The mice were infected by orogastric gavage with 150 TM embryonated eggs. Data are mean ± SD (Naïve WT n = 6, and KO n = 4, TM infected WT n = 9, and KO n = 13 mice) from 2 independent experiments). Statistical significance was computed by the two-tailed Student’s t-test. (PDF) [file pone.0211244.s006.pdf]

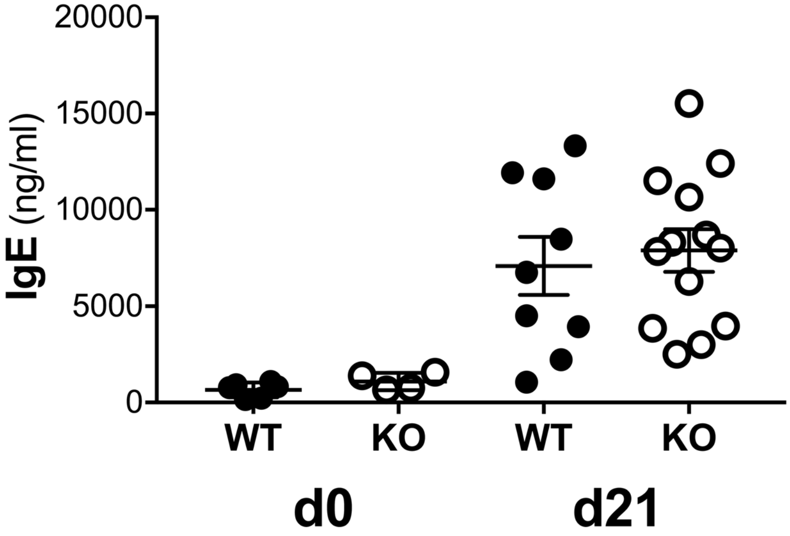

Fig S6. Serum IgE response to TM in Mina KO mice. Total serum IgE level from naive and TM infected WT and Mina KO mice at d21 p.i. The mice were infected by orogastric gavage with 150 TM embryonated eggs. Data are mean ± SD (Naïve WT n = 6, and KO n=4, Tm infected WT n=9, and KO n=13) mice from 2 independent experiments). Statistical significance was computed by the two-tailed Student's t-test.
