# Supplementary material for: Myc-induced nuclear antigen constrains a latent intestinal epithelial cell-intrinsic anthelmintic pathway
Source: PLoS One. 2019 Feb 26;14(2):e0211244. doi: 10.1371/journal.pone.0211244 (PMC6391002; doi:10.1371/journal.pone.0211244)
Supplement: S7 Fig — (A) Quantitative RT-PCR analysis of TSLP and IL33 mRNA in IECs from Mina KO and WT 21 dpi. Data are mean ± SD of n = 6 each of WT and KO for TSLP and WT n = 10, KO = 9 mice for IL33 from 2 independent experiments. TSLP ELISA was carried out on TM antigen stimulated mesenteric LN culture supernatants using a TSLP ELISA kit as described in methods. Data is from one of two independent experiments. (C) Cytokine analysis was performed for assessing IL17, IL13, IL4, IL10 using Milliplex cytokine analysis kit. Data are mean ± SEM (IL17; WT n = 8 and KO n = 10, IL13, WT n = 7 and KO n = 8, IL4; WT n = 9 and KO n = 7; IL10; WT n = 9 and KO = 10 mice; from 2 independent experiments). Statistical significance was computed by the two-tailed Student’s t-test. (PDF) [file pone.0211244.s007.pdf]

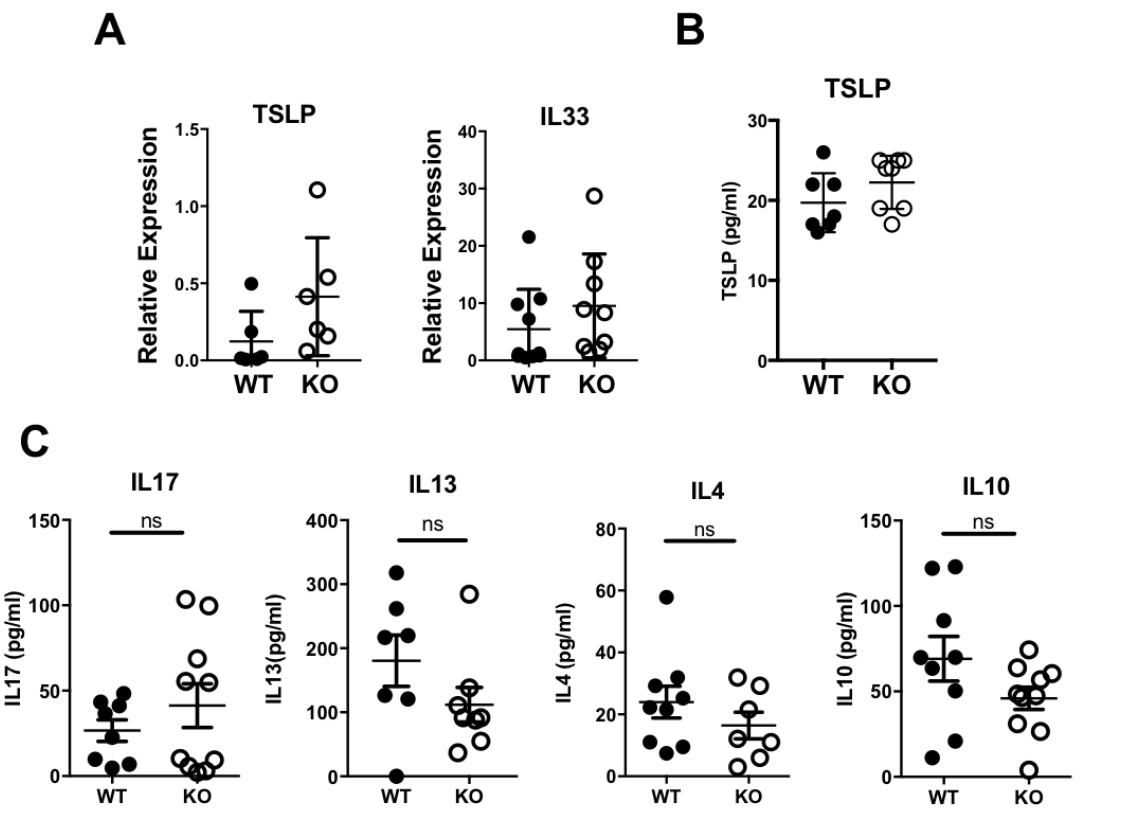

Fig S7. Th2 and Th17 response to TM in Mina KO mice. (A) Quantitative RT-PCR analysis of TSLP and IL33 mRNA in IECs from Mina KO and WT 21 dpi. Data are mean ± SD of n=6 each of WT and KO for TSLP and WT n=10, KO=9 mice for IL33 from 2 independent experiments. TSLP ELISA was carried out on TM antigen stimulated mesenteric LN culture supernatants using a TSLP ELISA kit as described in methods. Data is from one of two independent experiments. (C) Cytokine analysis was performed for assessing IL17, IL13, IL4, IL10 using Milliplex cytokine analysis kit. Data are mean ± SEM (IL17; WT n=8 and KO n=10, IL13, WT n=7 and KO n=8, IL4; WT n=9 and KO n=7; IL10; WT n=9 and KO=10 mice; from 2 independent experiments). Statistical significance was computed by the two-tailed Student’s t-test.
