# Supplementary material for: Myc-induced nuclear antigen constrains a latent intestinal epithelial cell-intrinsic anthelmintic pathway
Source: PLoS One. 2019 Feb 26;14(2):e0211244. doi: 10.1371/journal.pone.0211244 (PMC6391002; doi:10.1371/journal.pone.0211244)
Supplement: S8 Fig — Mina KO and WT littermate controls were differentiated under Th1 and Th2 conditions as described in methods. Shown are the mean ± SD (n = 3 mice for Th1 and n = 4 for Th2 from 1 of 2 representative experiments). Statistical significance was computed by the two-tailed Student’s t-test. (PDF) [file pone.0211244.s008.pdf]

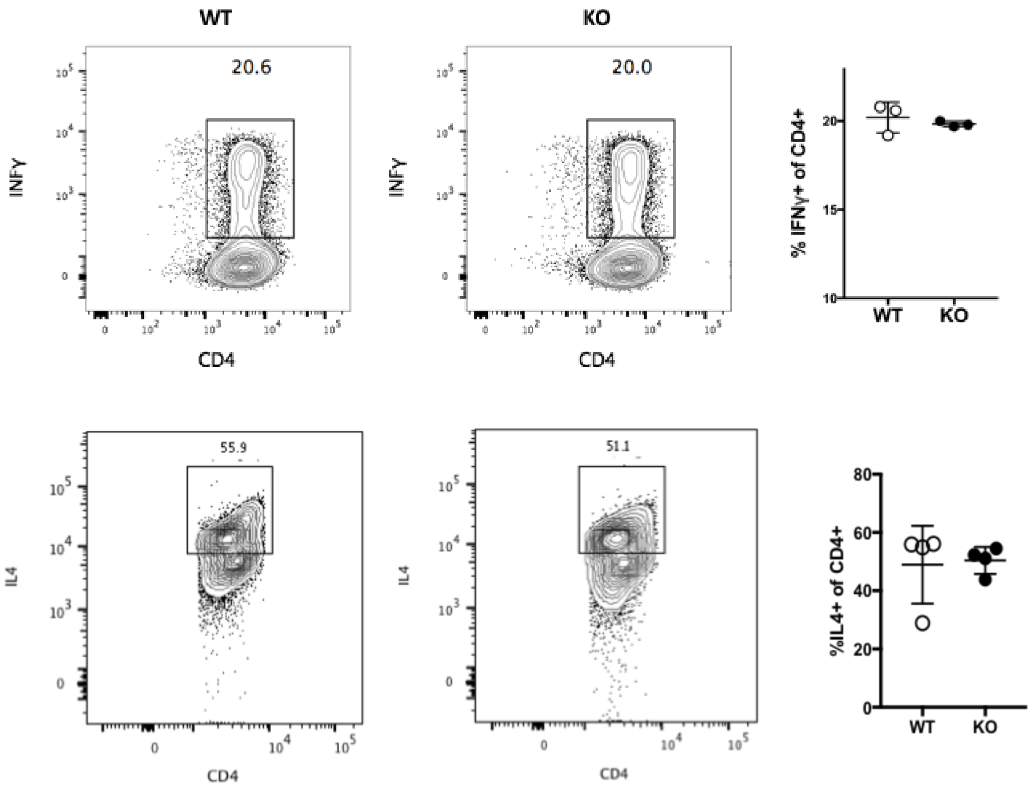

Fig S8. In vitro differentiation of Mina KO CD4 T cells. Mina KO and WT littermate controls were differentiated under Th1 and Th2 conditions as described in methods. Shown are the mean ± SD (n = 3 mice for Th1 and n=4 for Th2 from 1 of 2 representative experiments). Statistical significance was computed by the two-tailed Student’s t-test.
