# Supplementary material for: Myc-induced nuclear antigen constrains a latent intestinal epithelial cell-intrinsic anthelmintic pathway
Source: PLoS One. 2019 Feb 26;14(2):e0211244. doi: 10.1371/journal.pone.0211244 (PMC6391002; doi:10.1371/journal.pone.0211244)
Supplement: S9 Fig — Mina mRNA expression level in splenocytes and IECs from MinaΔIEC [VillinCre+::Mina(fl/fl)] and controls VillinCre+::Mina(+/+) and VillinCre-::Mina(fl/fl)] mice. Shown are the mean ± SEM (n = 11, 5 and 9 for Splenocytes and n = 7, 5, 8 for IECs respectively from 2 independent experiments). Statistical significance was computed by the two-tailed Student’s t-test. (PDF) [file pone.0211244.s009.pdf]

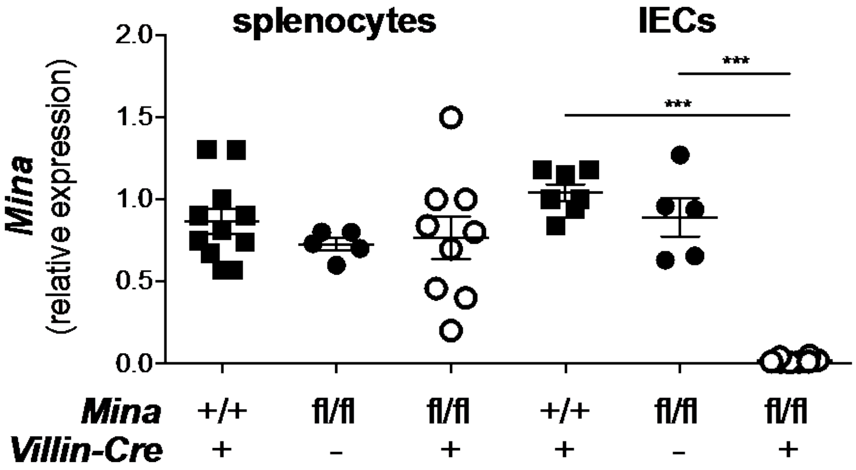

Fig S9. Splenic and IEC Mina expression in Mina$^{\Delta IEC}$ mice. Mina mRNA expression level in splenocytes and IECs from Mina$^{\Delta IEC}$ [VillinCre+::Mina(fl/fl)] and controls VillinCre+::Mina(+/+) and VillinCre-::Mina(fl/fl)] mice. Shown are the mean ± SEM (n = 11, 5 and 9 for Splenocytes and n=7,5,8 for IECs respectively from 2 independent experiments). Statistical significance was computed by the two-tailed Student's t-test.
