# Supplementary material for: Myc-induced nuclear antigen constrains a latent intestinal epithelial cell-intrinsic anthelmintic pathway
Source: PLoS One. 2019 Feb 26;14(2):e0211244. doi: 10.1371/journal.pone.0211244 (PMC6391002; doi:10.1371/journal.pone.0211244)
Supplement: S10 Fig — WT and Mina KO mice were infected by subcutaneous injection with 500 L3 Nippostrongylus brasiliensis larvae. At 7 dpi the mice were sacrificed and intestines were collected, dissected and resident larvae and adult worms were counted using a dissection microscope. Data are mean ± SD (n = 6 mice from one experiment). Statistical significance was computed by the Mann-Whitney test. (PDF) [file pone.0211244.s010.pdf]

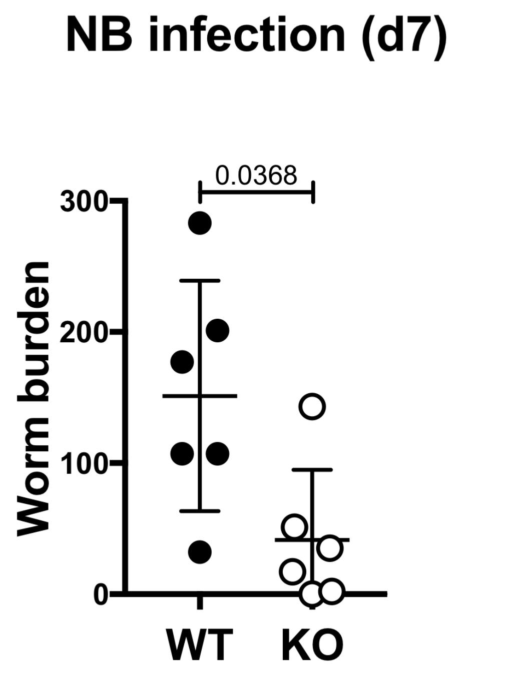

Fig. S10. Accelerated clearance of *Nippostrongylus brasiliensis* in the absence of Mina53. WT and Mina KO mice were infected by subcutaneous injection with 500 L3 *Nippostrongylus brasiliensis* larvae. At 7 dpi the mice were sacrificed, and intestines were collected, dissected and resident larvae and adult worms were counted using a dissection microscope. Data are mean ± SD (n=6 mice from one experiment). Statistical significance was computed by the Mann-Whitney test.
