# Supplementary material for: Myc-induced nuclear antigen constrains a latent intestinal epithelial cell-intrinsic anthelmintic pathway
Source: PLoS One. 2019 Feb 26;14(2):e0211244. doi: 10.1371/journal.pone.0211244 (PMC6391002; doi:10.1371/journal.pone.0211244)
Supplement: S11 Fig — Quantitative RT-PCR analysis of Rbp2 in 21 d.p.i. Mina KO and WT IECs. Data are mean ± SD (WT n = 8, and KO n = 5 mice from 2 independent experiments). Statistical significance was computed by the two-tailed Student’s t-test. (PDF) [file pone.0211244.s011.pdf]

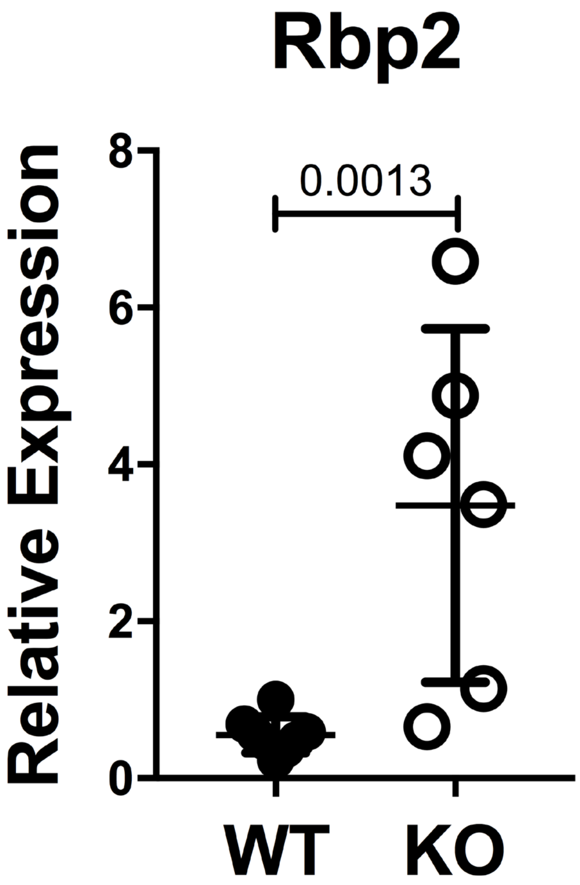

Fig S11. **Quantitative RT-PCR analysis of Rbp2.** Quantitative RT-PCR analysis of Rbp2 in 21 d.p.i. Mina KO and WT IECs. Data are mean ± SD (WT n =8, and KO n=5 mice from 2 independent experiments). Statistical significance was computed by the two-tailed Student's t-test.
