# Supplementary material for: Myc-induced nuclear antigen constrains a latent intestinal epithelial cell-intrinsic anthelmintic pathway
Source: PLoS One. 2019 Feb 26;14(2):e0211244. doi: 10.1371/journal.pone.0211244 (PMC6391002; doi:10.1371/journal.pone.0211244)
Supplement: S12 Fig — Cytotoxic activity of reduced (r-) α-defensins 5, 20, 21, 23 and 24 against L3 TM larvae was measured by lactate dehydrogenase release assay. Data are mean ± SD (n = 3 larvae), representative of 2–4 independent experiments. α-defensin 5 was positive in 3 of 4 experiments. Statistical significance was computed by two-tailed Student’s t-test. (PDF) [file pone.0211244.s012.pdf]

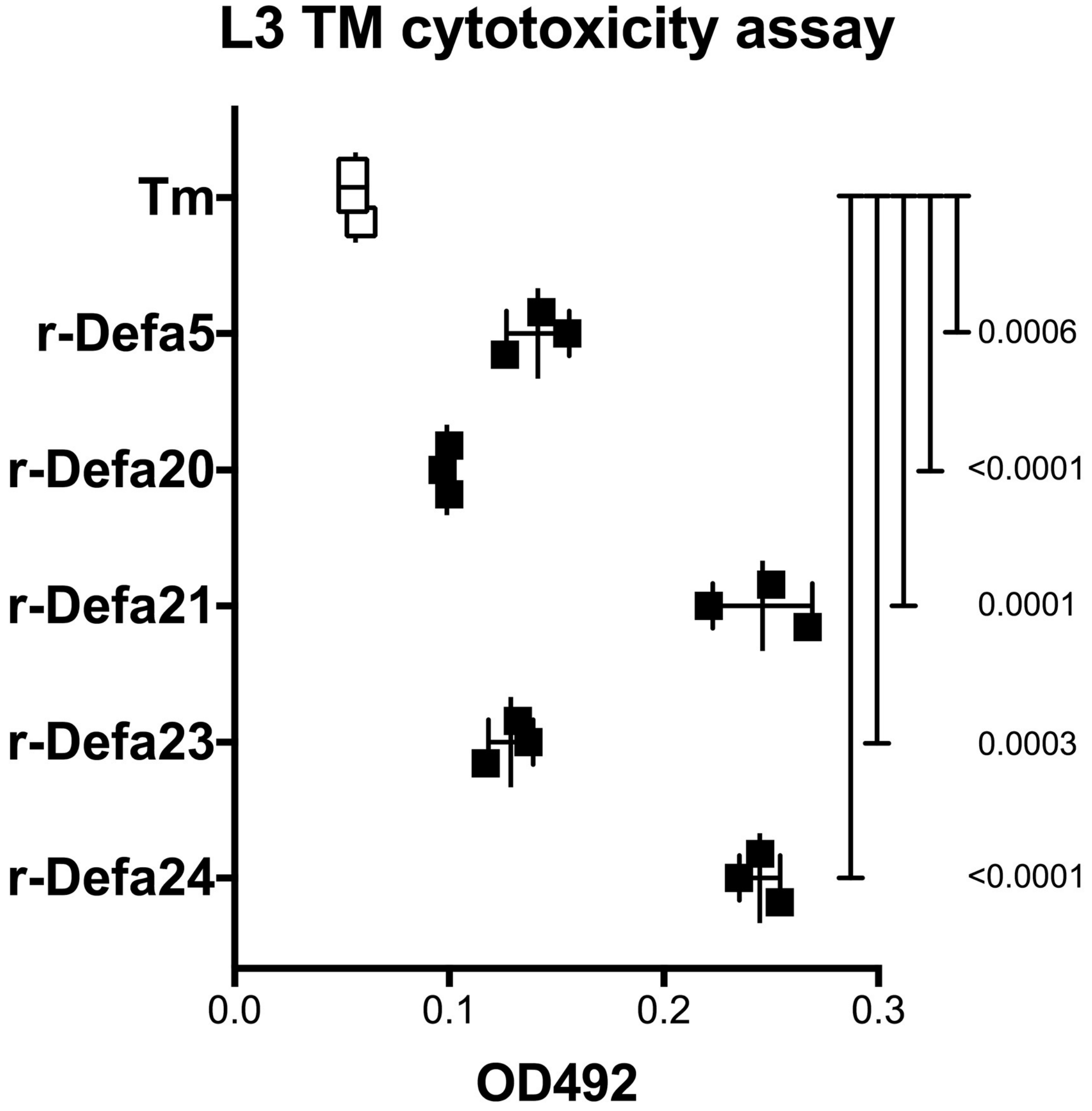

S12 Fig. Cytotoxic activity of α-defensins toward L3 TM larvae. Cytotoxic activity of reduced (r-) α-defensins 5, 20, 21, 23 and 24 against L3 TM larvae was measured by lactate dehydrogenase release assay. Data are mean ± SD (n =3 larvae), representative of 2-4 independent experiments. α-defensin 5 was positive in 3 of 4 experiments. Statistical significance was computed by two-tailed Student's t-test.
