# Supplementary material for: Myc-induced nuclear antigen constrains a latent intestinal epithelial cell-intrinsic anthelmintic pathway
Source: PLoS One. 2019 Feb 26;14(2):e0211244. doi: 10.1371/journal.pone.0211244 (PMC6391002; doi:10.1371/journal.pone.0211244)
Supplement: S13 Fig — Cytotoxic activity of reduced (r-) α-defensins 1, 2, 4, 5 and 6 against L3 TM larvae was measured by lactate dehydrogenase release assay. Data are mean ± SD (n = 3 larvae) in 1 experiment. Statistical significance was computed by the two-tailed Student’s t-test. (PDF) [file pone.0211244.s013.pdf]

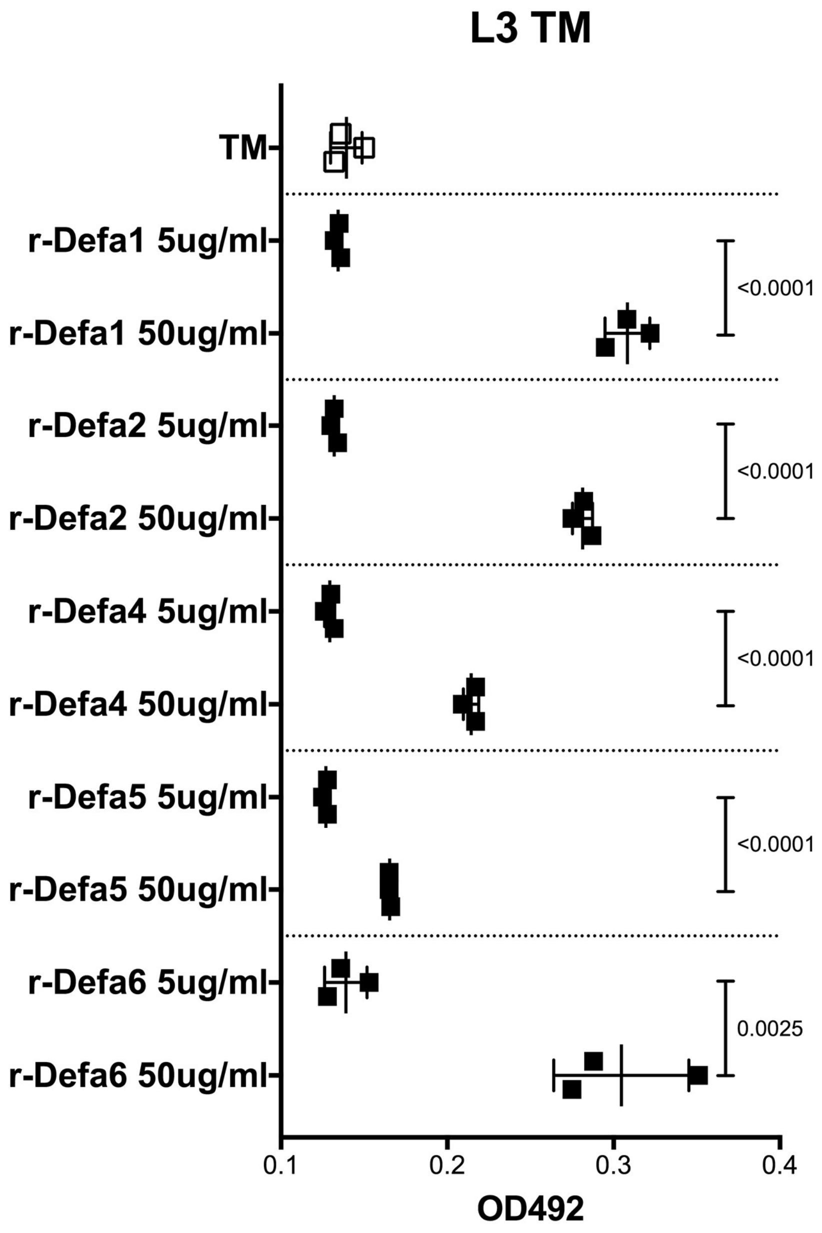

S13 Fig. Dose dependent cytotoxic activity of α-defensins toward L3 TM larvae. Cytotoxic activity of reduced (r-) α-defensins 1, 2, 4, 5 and 6 against L3 TM larvae was measured by lactate dehydrogenase release assay. Data are mean ± SD (n = 3 larvae) in 1 experiment. Statistical significance was computed by the two-tailed Student’s t-test.
