# Supplementary material for: Myc-induced nuclear antigen constrains a latent intestinal epithelial cell-intrinsic anthelmintic pathway
Source: PLoS One. 2019 Feb 26;14(2):e0211244. doi: 10.1371/journal.pone.0211244 (PMC6391002; doi:10.1371/journal.pone.0211244)
Supplement: S14 Fig — Cytotoxic activity of reduced (r-) and oxidized (o-) α-defensins 1 and 4 against L3 TM larvae was measured by lactate dehydrogenase release assay. Data are mean ± SD (n = 3 larvae), representative of 2 independent experiments. Statistical significance was computed by the two-tailed Student’s t-test. (PDF) [file pone.0211244.s014.pdf]

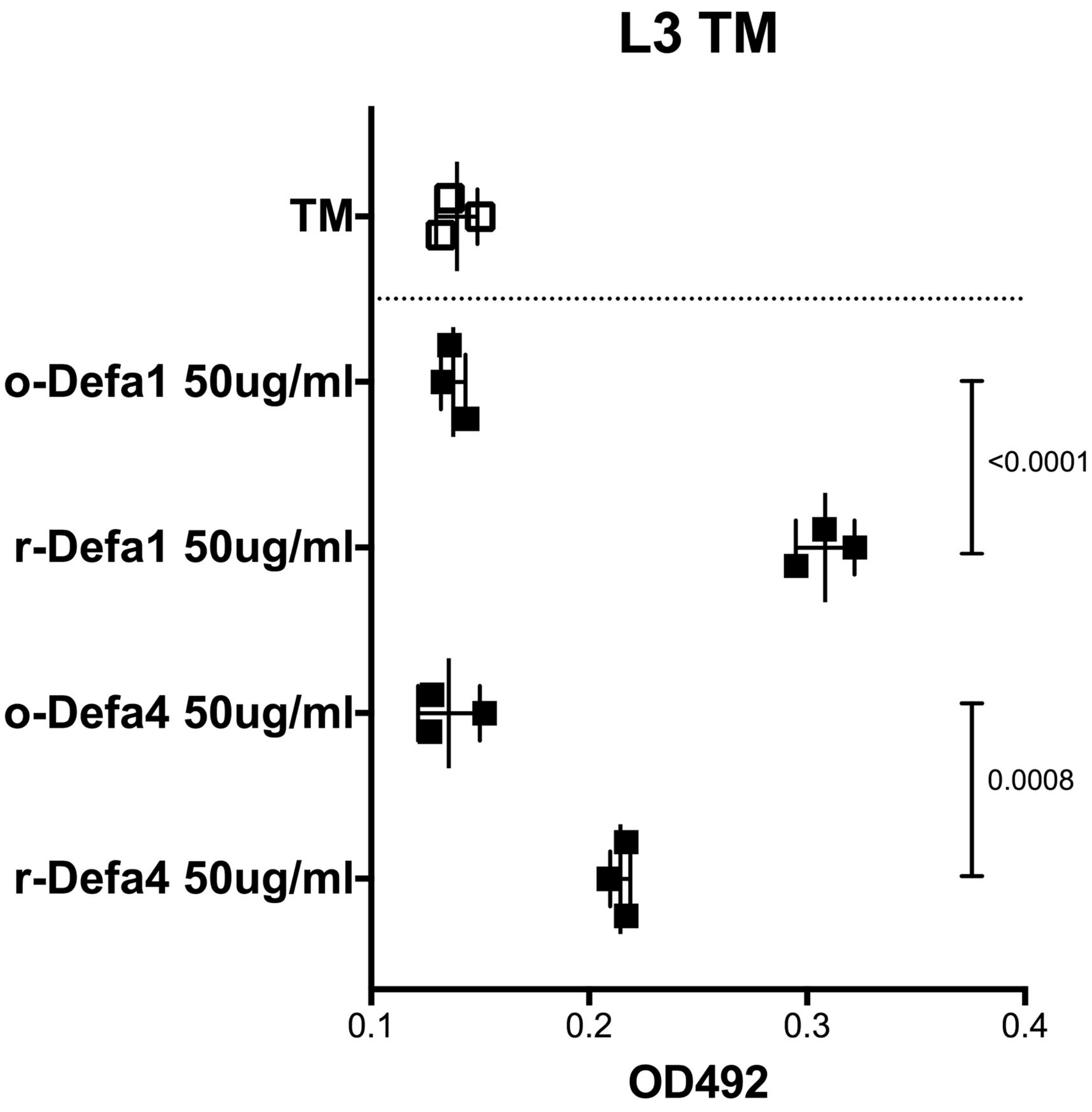

S14 Fig. Reduced but not oxidized α-defensins exhibit cytotoxic activity toward L3 TM larvae. Cytotoxic activity of reduced (r-) and oxidized (o-) α-defensins 1 and 4 against L3 TM larvae was measured by lactate dehydrogenase release assay. Data are mean ± SD (n = 3 larvae), representative of 2 independent experiments. Statistical significance was computed by the two-tailed Student's t-test.
