# Supplementary material for: Myc-induced nuclear antigen constrains a latent intestinal epithelial cell-intrinsic anthelmintic pathway
Source: PLoS One. 2019 Feb 26;14(2):e0211244. doi: 10.1371/journal.pone.0211244 (PMC6391002; doi:10.1371/journal.pone.0211244)
Supplement: S1 Table — Mina KO and WT littermate control mesenteric lymph node, peripheral lymph node, spleen and thymic cells stained for various cell surface and intracellular markers as described in the Material and Methods and analyzed for the percentage of different cell subsets. Data are mean ± SD (from 2 independent experiments). Statistical significance was computed by the Mann Whitney test. (PDF) [file pone.0211244.s015.pdf]

S1 Table. Lymphoid and myeloid immunophenotypic analysis of Mina KO mice.

Mina KO and WT littermate control mesenteric lymph node, peripheral lymph node, spleen and thymic cells stained for various cell surface and intracellular markers as described in the Material and Methods and analyzed for the percentage of different cell subsets. Data are mean ± SD (from 2 independent experiments). Statistical significance was computed by the Mann Whitney test.

Subset	markers	Tissue	genotype	N	Mean	SD	sig dif?	p value
double negative	CD4- CD8-	Thymus	WT	6	3.163	0.2617	No	0.5491
			KO	9	3.477	0.2703		
CD8 single positive	CD4- CD8+		WT	6	3.19	0.6675	No	0.2949
			KO	7	2.77	0.2483		
CD4 single positive	CD4+ CD8-		WT	6	10.23	1.805	No	0.9264
			KO	8	9.633	0.5483		
double positive	CD4+ CD8+		WT	6	83.42	2.476	No	0.5491
			KO	8	83.94	1.154		
CD4 T cell	CD4+CD8-	mesenteric lymph node	WT	4	37.18	0.7932	No	0.0952
			KO	6	34.57	1.992		
		peripheral lymph node	WT	6	32.42	4.832	No	0.3852
			KO	7	35.64	3.024		
		spleen	WT	6	25.12	2.723	No	0.2824
			KO	8	23.01	3.507		
CD8 T cell	CD8+CD4-	mesenteric lymph node	WT	5	26.34	6.369	No	0.4286
			KO	6	29.22	4.148		
		peripheral lymph node	WT	6	38.33	2.86	No	0.366
			KO	7	36.99	2.443		
		spleen	WT	6	13.97	1.118	No	0.345
			KO	8	15.03	2.141		
B cell	B220+	mesenteric lymph node	WT	4	30.48	1.282	Yes	0.0159
			KO	5	33.38	0.7014		
		peripheral lymph node	WT	6	25.98	8.066	No	0.9452
			KO	7	23.64	4.984		
		spleen	WT	6	51.75	4.057	No	0.9497
			KO	8	51.83	5.963		
macrophage	B220- CD11b+CD11c- Gr1-	mesenteric lymph node	WT	5	0.138	0.01924	No	0.2063
			KO	5	0.122	0.01483		
		peripheral	WT	6	0.148	0.04143	No	0.1457

		lymph node	KO	7	0.112	0.03663		
		spleen	WT	6	1.467	0.2391	No	0.3623
			KO	8	1.613	0.2295		
CD8 positive DC	B220-CD11b-CD11c+CD8+	mesenteric lymph node	WT	5	0.145	0.05297	Yes	0.0216
			KO	6	0.282	0.1602		
		peripheral lymph node	WT	6	0.05933	0.01787	No	0.9732
			KO	7	0.05857	0.01631		
		spleen	WT	6	0.275	0.0437	No	0.5951
			KO	8	0.26	0.08832		
CD8 negative DC	B220-CD11b-CD11c+CD8-	mesenteric lymph node	WT	5	0.352	0.1103	No	0.2662
			KO	6	0.43	0.174		
		peripheral lymph node	WT	6	0.1433	0.03141	No	0.3479
			KO	7	0.1757	0.05318		
		spleen	WT	6	1.793	0.2214	No	0.4692
			KO	8	1.646	0.3927		
plasmacytoid DC	B220+CD11b-CD11c+F4/80-Gr1+	mesenteric lymph node	WT	5	0.0144	0.004393	No	0.2063
			KO	5	0.019	0.005916		
		peripheral lymph node	WT	6	0.02283	0.006014	No	0.5583
			KO	7	0.02057	0.008142		
		spleen	WT	6	0.004028	0.002627	No	0.2403
			KO	6	0.002543	0.001321		
