# Supplementary material for: Myc-induced nuclear antigen constrains a latent intestinal epithelial cell-intrinsic anthelmintic pathway
Source: PLoS One. 2019 Feb 26;14(2):e0211244. doi: 10.1371/journal.pone.0211244 (PMC6391002; doi:10.1371/journal.pone.0211244)
Supplement: S2 Table — Mature peptide sequences are in bold font. (PDF) [file pone.0211244.s016.pdf]

**S2 Table. RNAseq genes with expression greater than 2-fold higher in infected Mina-deficient as compared to uninfected control IECs and with p < 0.01 and that were confirmed by qRT-PCR.**

Mature peptide sequences are in bold font.

| gene name | cDNA sequence | protein sequence |
|---|---|---|
| **Defa5** | ATGAAGACATTTGTCCTCCTCTCTGCCCTTGTCCTGCTGGCCTTCCAGGCCCAGGCTGATCCT<br>ATCCACAAAACAGATGAAGAGACTAATACTGAGGAGCAGCCAGGGGAAGAGGACCAGGCTGT<br>GTCTATCTCCTTTGGAGGCCAAGAAGGGTCTGCTCTTCATGAAGAATTGTCAAAAAAGCTGATA<br>TGCTATTGTAGAATAAGAGGCTGCAAAAGAAGAGAACGCGTTTTTGGGACCTGCAGAAATCTTT<br>TTTTAACTTTCGTATTCTGCTGCAGCTGA | MKTFVLLSALVLLAFQAQAD<br>PIHKTDEETNTEEQPGEEDQ<br>AVSISFGGQEGSALHEE**LSK**<br>**KLICYCRIRGCKRRERVFGT**<br>**CRNLFLTFVFCCS** |
| **Defa20** | ATGAAGACACTTGTCCTCCTCTCTGCCCTCGTCCTGCTGGCCTTCCAGGTCCAGGCTGATCCT<br>ATCCAAAACACAGATGAGGAGACTAATACTGAGGAGCAGCCAGGGGAGGAGGACCAGGCTGT<br>GTCTGTCTCCTTTGGAGACCCAGAAGGATCTGCTCTTCATGAAAAATCGTCGAGAGATCTGATA<br>TGCTATTGTAGAAAAGGAGGCTGCAATAGAGGAGAACAAGTTTATGGGACCTGCTCAGGACGA<br>CTTTTGTTCTGCTGCCGCCGCCGCCACCGCCACTGA | MKTLVLLSALVLLAFQVQAD<br>PIQNTDEETNTEEQPGEED<br>QAVSVSFGDPEGSALHEK**S**<br>**SRDLICYCRKGGCNRGEQV**<br>**YGTCSGRLLFCCRRRHRH** |
| **Defa21** | ATGAAGACACTTGTCCTCCTCTCTGCCCTCATCCTGCTGGCCTACCAGGTCCAGACTGATCCT<br>ATCCAAAACACAGATGAAGAGACTAATACTGAGGAGCAGCCAGGGGAAGATGACCAGGCTGT<br>GTCTGTCTCCTTTGGAGGCCAAGAAGGATCTGCTCTTCATGAAAAATTGTCGAGAGATCTGATC<br>TGCCTTTGTAGAAATCGTCGCTGCAATAGAGGAGAACTATTTTATGGGACCTGCGCAGGACCT<br>TTTTTGCGCTGCTGCCGCCGCCGCCGCTGA | MKTLVLLSALILLAYQVQTDP<br>IQNTDEETNTEEQPGEDDQ<br>AVSVSFGGQEGSALHEK**LS**<br>**RDLICLCRNRRCNRGELFY**<br>**GTCAGPFLRCCRRRR** |
| **Defa22** | ATGAAGACACTTGTCCTCCTCTCTGCCCTCATCCTGCTGGCCTACCAGGTCCAGACTGATCCT<br>ATCCAAAACACAGATGAAGAGACTAATACTGAGGAGCAGCCAGGGGAAGAGGACCAGGCTGT<br>GTCTGTCTCCTTTGGAGGCCAAGAAGGATCTGCTCTTCATGAAAAATTGTCGAGAGATCTGATC<br>TGCCTTTGTAGAAAACGTCGCTGCAATAGAGGAGAACTATTTTATGGGACCTGCGCAGGACCT<br>TTTTTGCGCTGCTGCCGCCGCCGCCGCTGA | MKTLVLLSALILLAYQVQTDP<br>IQNTDEETNTEEQPGEEDQ<br>AVSVSFGGQEGSALHEK**LS**<br>**RDLICLCRKRRCNRGELFY**<br>**GTCAGPFLRCCRRRR** |
| **Defa23** | ATGAAGACACTAGTCCTCCTCTCTGCCCTCATCCTGCTGGCCTTCCAGGTCCAGGCTGATCCT<br>ATCCAAAACACAGATGAAGAGACTAAAACTGAGGAGCAGCCAGGGAAAGAGGACCAGGCTGT<br>GTCTGTCTCTTTTGGAGACCCAGAAGGCTCTTCTCTTCAAGAGGAATCGTTGAGAGATCTGGTA<br>TGCTATTGTAGAACAAGAGGCTGCAAAAGAAGAGAACGCATGAATGGGACCTGCAGAAAGGGT<br>CATTTAATATACACGCTCTGCTGTCGCTGA | MKTLVLLSALILLAFQVQADP<br>IQNTDEETKTEEQPGKEDQ<br>AVSVSFGDPEGSSLQEES**L**<br>**RDLVCYCRTRGCKRRERM**<br>**NGTCRKGHLIYTLCCR** |
| **Defa24** | ATGAAGACACTAATCCTCCTCTCTGCCCTCGTCCTGCTGGCCTTCCAGGTCCAGGCTGATCCT<br>ATCCAAAACACAGATGAAGAGACTAAAACTGAGGAGCAGCCAGGGGAAGAGGACCAGGCTGT<br>GTCTGTCTCTTTTGGAGACCCAGAAGGCTCTTCTCTTCAAGAGGAATCGTTGAGAGATCTGGTA<br>TGCTATTGTAGAGCAAGAGGCTGCAAAGGAAGAGAACGCATGAATGGGACCTGCAGTAAGGG<br>TCATTTAATGTACATGCTCTGCTGTCGCTGA | MKTLILLSALVLLAFQVQADP<br>IQNTDEETKTEEQPGEEDQ<br>AVSVSFGDPEGSSLQEES**L**<br>**RDLVCYCRARGCKGRERM**<br>**NGTCSKGHLMYMLCCR** |
| **Rbp2** | | |
