# Supplementary material for: Myc-induced nuclear antigen constrains a latent intestinal epithelial cell-intrinsic anthelmintic pathway
Source: PLoS One. 2019 Feb 26;14(2):e0211244. doi: 10.1371/journal.pone.0211244 (PMC6391002; doi:10.1371/journal.pone.0211244)
Supplement: S3 Table — (PDF) [file pone.0211244.s017.pdf]

S3 Table. Cytokines and antibodies.

Flow cytometry

Target	Conjugation	Clone	Source	Catalog number
CD11b	APC	M1/70	eBioscience	17-0112-81
CD11c	APC/Cy7	N418	Biolegend	117324
CD45R (B220)	FITC	RA3-6B2	eBioscience	11-0452-82
Ly6G (Gr1)	PE	RB6-8C5	eBioscience	12-7041-82
F4/80	FITC	BM8	eBioscience	53-4801-82
CD4	Pacific Blue	RM4-5	eBioscience	48-0042-82
CD8a	PerCP/Cy5.5	53-6.7	eBioscience	45-0081-80
CD8a	PE/Cy7	53-6.7	Biolegend	100722
CD45, Ly-5	APC	30-F11	eBioscience	17-0451-82
CD236 (EPCAM)	PE	G8.8	eBioscience	12-5791-82
IL4	PE	11B11	eBioscience	12-7041-81
IFNγ	APC	XMG1.2	eBioscience	17-7311-82
CD16/CD32	none	2.4G2	BD	553144
LIVE/DEAD® Fixable Aqua Dead Cell Stain Kit	none	-	Thermofisher	L34957

ELISA

Target	Conjugation	Clone	Host	Isotype	Source	Catalog number
isotype control Ab	none	polyclonal	mouse	IgE, k	BD	557080
IgE	none	R35-72	rat	IgG1, k	BD	553413
IgG (H&L)	AP	polyclonal	goat or donkey	-	Promega	S372B
IgG1	biotin	A85-1	rat	IgG1, k	BD	553441
IgG2c	biotin	R19-15	rat	IgG1, k	BD	553388

Cell culture

Target	Clone	Source	Catalog number
CD28	37.51	eBioscience	16-0281-85
CD3e	145-2C11	eBioscience	16-0031-85
