# Supplementary material for: Myc-induced nuclear antigen constrains a latent intestinal epithelial cell-intrinsic anthelmintic pathway
Source: PLoS One. 2019 Feb 26;14(2):e0211244. doi: 10.1371/journal.pone.0211244 (PMC6391002; doi:10.1371/journal.pone.0211244)
Supplement: S4 Table — (PDF) [file pone.0211244.s018.pdf]

S4 Table. PCR primers and probes.

qRT-PCR primers and probes	
**Gene**	**Primer /Taqman**
Defa5	Mm00651548_g1
Defa20	Mm00842045_g1
Defa21	Mm04206099_gH
Chga	Mm00514341_m1
Sox9	Mm00448840_m1
Lgr5	Mm00438890_m1
Lyz1	Mm00657323_m1
Mina	Mm01175641_m1
Muc2	Mm01276696_m1
Gapdh	Mm99999915_g1
